# A Brg1-Rme1 circuit in *Candida albicans* hyphal gene regulation

**DOI:** 10.1128/mbio.01872-24

**Published:** 2024-07-30

**Authors:** Min-Ju Kim, Max Cravener, Norma Solis, Scott G. Filler, Aaron P. Mitchell

**Affiliations:** 1 Department of Microbiology, University of Georgia, Athens, Georgia, USA; 2 Lundquist Institute for Biomedical Innovation at Harbor-UCLA Medical Center, Torrance, California, USA; 3 David Geffen School of Medicine at UCLA, Los Angeles, California, USA; The University of Texas Health Science Center at Houston, Houston, Texas, USA

**Keywords:** biofilms, *Candida albicans*, transcriptional regulation, hypoxia, hyphal development

## Abstract

Major *Candida albicans* virulence traits include its ability to make hyphae, to produce a biofilm, and to damage host cells. These traits depend upon expression of hypha-associated genes. A gene expression comparison among clinical isolates suggested that transcription factor Rme1*,* established by previous studies to be a positive regulator of chlamydospore formation, may also be a negative regulator of hypha-associated genes. Engineered *RME1* overexpression supported this hypothesis, but no relevant *rme1*Δ/Δ mutant phenotype was detected. We reasoned that Rme1 may function within a specific regulatory pathway. This idea was supported by our finding that an *rme1*Δ/Δ mutation relieves the need for biofilm regulator Brg1 in biofilm formation. The impact of the *rme1*Δ/Δ mutation is most prominent under static or “biofilm-like” growth conditions. RNA sequencing (RNA-seq) of cells grown under biofilm-like conditions indicates that Brg1 activates hypha-associated genes indirectly via repression of *RME1*: hypha-associated gene expression levels are substantially reduced in a *brg1*Δ/Δ mutant and partially restored in a *brg1*Δ/Δ *rme1*Δ/Δ double mutant. An *rme1*Δ/Δ mutation does not simply bypass Brg1, because iron homeostasis genes depend upon Brg1 regardless of Rme1. Rme1 thus connects Brg1 to the targets relevant to hypha and biofilm formation under biofilm growth conditions.

**IMPORTANCE:**

*Candida albicans* is a major fungal pathogen of humans, and its ability to grow as a surface-associated biofilm on implanted devices is a common cause of infection. Here, we describe a new regulator of biofilm formation, *RME1*, whose activity is most prominent under biofilm-like growth conditions.

## INTRODUCTION

For many pathogens, traits that impact virulence vary among isolates (1–6). Variation may reflect adaptations that favor distinct niches or infection routes, impact of antimicrobial therapy, or other factors. Correlations between virulence traits and potential causal factors, such as mutations or gene expression features, can reveal new genes or circuits that modulate infection ability.

Our focus is *Candida albicans*, a prominent fungal pathogen that also exists benignly in the human genitourinary and gastrointestinal tracts (7, 8). *C. albicans* has many well-recognized virulence traits, including the ability to grow as filamentous hyphal cells, to damage host cells, and to produce a biofilm community (9, 10). Virulence traits vary tremendously among clinical isolates. In a few cases, causal mutations that drive variation have been identified (11, 12), but for most strains, the causal mutation or mutations are uncertain. Genetic background effects often reflect a combination of several mutations, each of which has a small effect size (13–15). Because of the many sequence differences among *C. albicans* isolates (12, 16), it has been challenging to find causal alleles behind virulence trait variation.

Strain variation in virulence traits has nonetheless been used successfully for functional gene discovery. The general approach has been to correlate gene expression differences and phenotypic differences as a strategy to identify new candidate genes that may govern the phenotype. Success of such a genome-wide approach was illustrated by the pioneering work of Kvitek et al. on *S. cerevisiae* (17). They used gene expression differences among diverse *S. cerevisiae* isolates to identify genes that function in tolerance to environmental stresses. For *C. albicans*, this type of approach was first applied (to our knowledge) by Wang et al. (18), in which RNA-seq data from 21 *C*. *albicans* clinical isolates were used to assemble co-expression networks associated with diverse traits. For two traits, “gray” cell growth and filamentation, several novel network genes were functionally validated by deletion mutant analysis (18). The approach was also applied by Do et al. (19) to identify genes that modulate the gene expression impact of the master cell type regulator Efg1, revealing that the Efg1 antagonist Wor1 can act in conjunction with Efg1 to promote biofilm formation. Thus, the gene expression-phenotype correlation among clinical isolates offers a powerful approach for gene discovery.

Here, we have used gene expression variation among clinical isolates to explore the determinants of host epithelial cell damage by *C. albicans*. Damage is mediated by Candidalysin, a toxic peptide processed from the Ece1 gene product that is required for virulence (20). Damage also depends upon the *C. albicans* surface adhesin Als3, which induces endocytosis of the fungal cells and enables focused delivery of Candidalysin (21). Both *ECE1* and *ALS3* are expressed at much higher levels in hyphal cells than in yeast cells and are considered hypha-associated genes (22). Epithelial cell damage triggers an inflammatory response that ultimately clears infection (23). In fact, strains with defects in damage have improved ability to colonize the oral mucosa (24). Because there are diverse roles of host cell damage ability—impairing commensalism or favoring virulence—it seemed reasonable that damage ability may vary among clinical *C. albicans* isolates. Our study exploits such variation to define *RME1* as a new regulator of hypha-associated genes.

*RME1* specifies a C_2_H_2_ zinc finger transcription factor. It functions as a positive regulator of asexual chlamydospore production in *C. albicans* and related species (25). However, to our knowledge, Rme1 has not been shown to affect virulence traits. Our studies connect Rme1 functionally to Brg1, which is considered a biofilm master regulator (26). Brg1 specifies a GATA-type transcription factor that is required for hypha-associated gene expression (27, 28). Brg1 does not bind directly to 5′ regions of many hypha-associated genes that encode effectors (e.g., *ALS3*, *ECE1*, *HGC1*, *HWP1*, and *HYR1*) but instead binds to 5′ regions of regulatory genes (e.g., *BCR1*, *NRG1*, and *UME6*) (28). Thus, Brg1 governs hypha-associated gene expression indirectly. Prior studies show that *NRG1* and *UME6* are major mediators of Brg1 impact on hypha-associated genes (29). Our results here indicate that Rme1 also functions downstream of Brg1 and that its impact is most prominent in a biofilm environment.

## RESULTS AND DISCUSSION

### Correlation between gene expression and host cell damage

**Fig 1:**
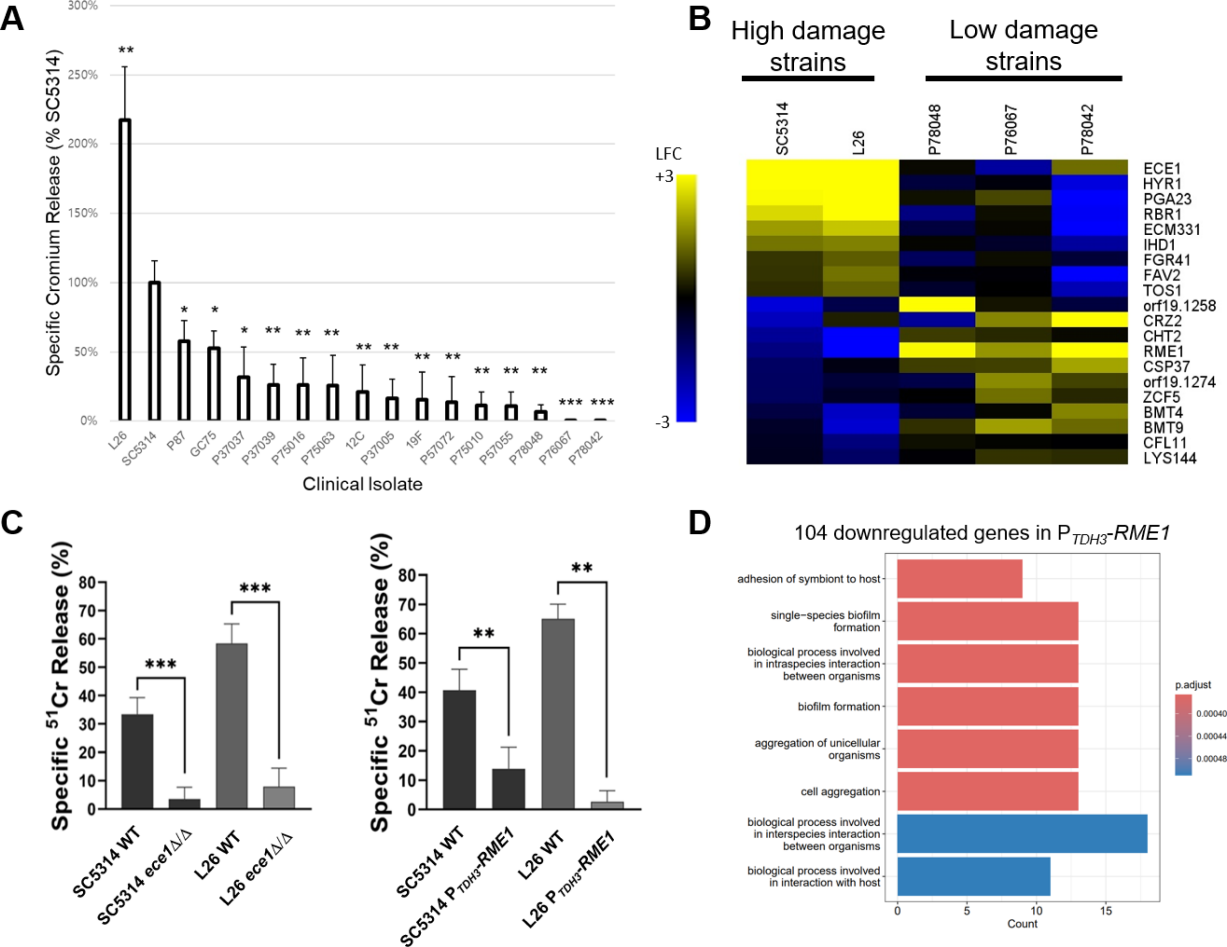
*RME1* and epithelial cell damage. (A) Seventeen clinical isolates were assayed for ability to damage OKF6/TERT-2 oral epithelial cells (21). Results are presented as the % damage relative to that caused by reference strain SC5314. Statistical analysis was conducted using an unpaired *t*-test, comparing to SC5314. Asterisks denote statistically significant differences. *P value < 0.05, **P value < 0.01, and ***P value < 0.001. (**B**)Gene expression profiles that correlate with damage ability were identified by clustering RNA-seq data sets of Cravener et al. (30) (*C. albicans* cells cultured in RPMI + 10% FBS at 37°C for 4 hours with vigorous shaking). Expression data for two high-damage strains (SC5314 and L26) and three low-damage strains (P76067, P78042, and P78048) were used. The analysis yielded 507 correlated/anticorrelated genes (Table S1). The 20 genes shown in the heatmap were prioritized because they encode surface/secreted proteins or transcription factors and had a large expression difference between high- and low-damage strains. The scale is yellow (log_2_ fold change from species median of +3) to blue (log_2_ fold change from species median of −3). (**C**) Overexpression mutants or deletion mutants of the 20 prioritized genes were constructed in the high-damage SC5314 and L26 backgrounds and assayed for epithelial cell damage ability. Damage defects were evident in the *ece1*Δ/Δ and P*_TDH3_-RME1* mutants. Statistical analysis was conducted using one-way analysis of variance (ANOVA), and asterisks denote statistically significant differences. **P value < 0.01 and ***P value < 0.001. (**D**) RNA-seq analysis for SC5314 and its P*_TDH3_-RME1* overexpression derivative (Table S2) yielded 104 genes significantly downregulated in the P*_TDH3_-RME1* strain. Gene ontology (GO) term enrichment for these genes is shown.

We assayed 17 clinical isolates, including reference strain SC5314, for the ability to damage OKF6/TERT-2 oral epithelial cells (21). Damage ability varied quantitively (Fig. 1A): two strains produced high damage levels (L26 and SC5314), two strains produced no detectable damage (P76067 and P78042), and the remaining 13 strains produced low or intermediate damage levels. Damage ability of each strain was significantly different from that of SC5314. To identify *C. albicans* genes that may mediate epithelial cell damage ability, we identified genes whose expression levels correlated with epithelial cell damage. Expression levels were based on the RNA sequencing (RNA-seq) data sets of Cravener et al. (30), in which cells were cultured in RPMI + 10% fetal bovine serum (FBS) at 37°C for 4 hours with vigorous shaking before RNA extraction. Expression profiles for two high-damage strains (SC5314 and L26) and three low-damage strains (P76067, P78042, and P78048) were then clustered using a self-organizing tree algorithm. This analysis yielded 507 genes that were correlated or anticorrelated with epithelial cell damage (Table S1). We prioritized a subset of genes for functional analysis based on three criteria: (i) their predicted products were surface or secreted proteins, which may have a direct role in host cell interaction; (ii) alternatively, their predicted products were transcription factors, which may govern expression of a set of host interaction genes; and (iii) their difference in expression between high- and low-damage strains was large, increasing confidence that their expression difference may cause a prominent phenotypic difference. These considerations yielded 20 genes for functional analysis (Fig. 1B).

### Assessment of mutant epithelial damage phenotypes

We hypothesized that genes that were more highly expressed in high-damage than low-damage strains may have a positive role in damage. Therefore, we predicted that deletion mutants of these genes in high-damage strains should have reduced damage ability. We tested this hypothesis with homozygous deletion mutants for *ECE1*, *HYR1*, *PGA23*, *RBR1*, *ECM331*, *IHD1*, *FGR41*, *FAV2*, and *TOS1*. Similarly, we hypothesized that genes more weakly expressed in high-damage than low-damage strains may have a negative role in damage. We tested this hypothesis with *TDH3* promoter fusions to *orf19.1258*, *CRZ2*, *CHT2*, *RME1*, *CSP37*, *orf19.1274*, *ZCF5*, *BMT4*, *BMT9*, *CFL11*, and *LYS144* to create overexpression alleles. All mutant strains in the SC5314 and L26 backgrounds were then assayed for epithelial cell damage ability. Epithelial cell damage for most mutants was comparable to their respective wild-type strain. However, damage was reduced in the *ece1*Δ/Δ mutants (Fig. 1C), as expected from the well-established role of Ece1 in cell damage (9, 20). Damage was also reduced in the P*_TDH3_-RME1* mutant strains (Fig. 1C). This result supported the hypothesis that Rme1 has a negative role in epithelial cell damage.

### Impact of Rme1 overexpression

To elucidate Rme1 function in host damage, we conducted RNA-seq analysis for SC5314 and its derived P*_TDH3_-RME1* overexpression strain (Table S2). The RNA-seq data showed that P*_TDH3_-RME1* increases *RME1* RNA levels by ~500-fold in RPMI + 10% FBS. Increased *RME1* expression altered expression of 568 genes (log_2_ fold change > 1; adjusted *P* value < 0.05 [Table S2]). The 464 Rme1-activated genes showed enrichment for GO terms related to carbohydrate metabolism and arginine biosynthesis. The 104 *RME1*-repressed genes showed enrichment for GO terms related to biofilm formation and host interaction (Fig. 1D). In fact, *ECE1* and *ALS3* were downregulated in the P*_TDH3_-RME1* strain (Table S2). Reduced expression of *ECE1* and *ALS3* provides a simple explanation for the host cell damage defect caused by P*_TDH3_-RME1*.

**Fig 2:**
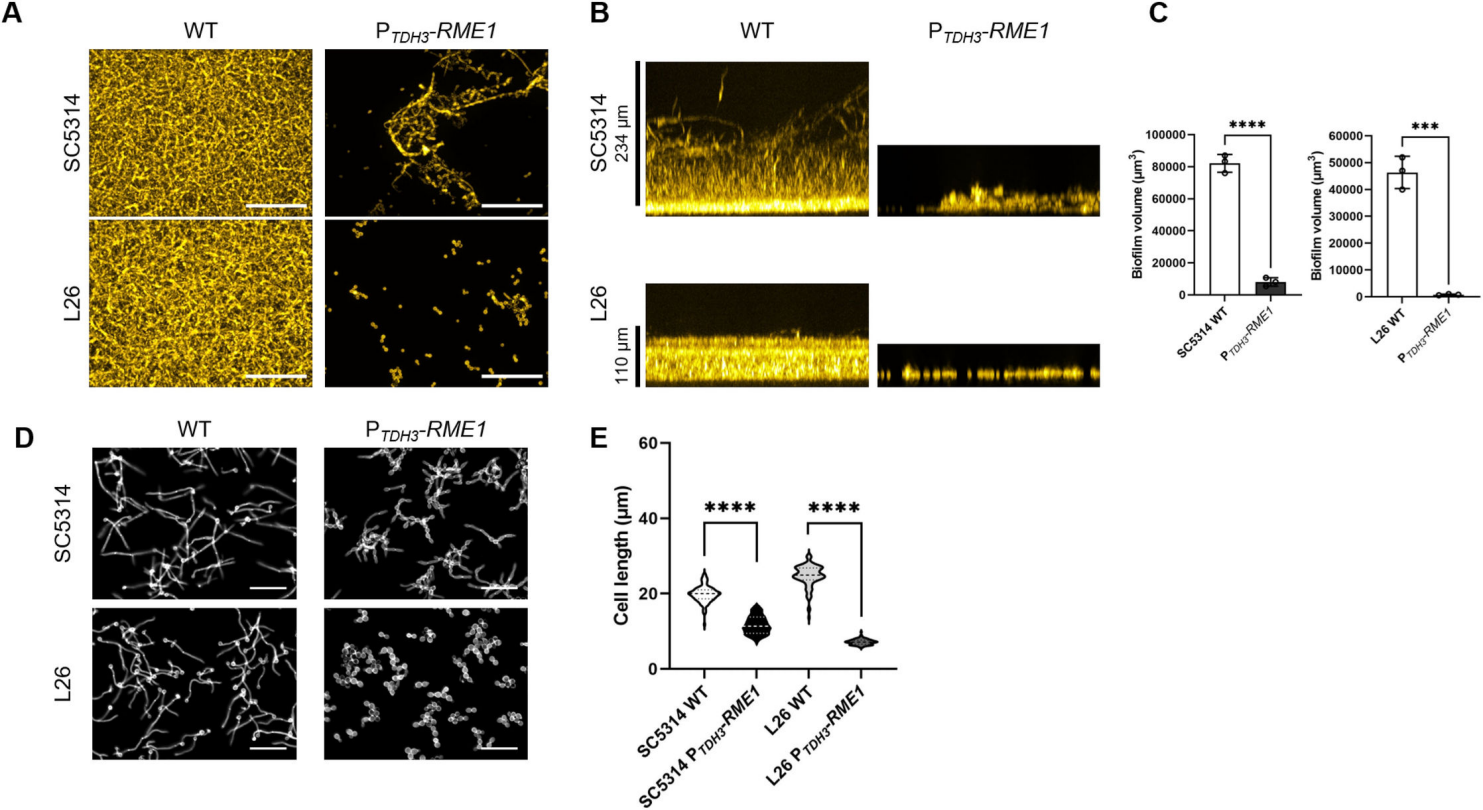
Impact of *RME1* overexpression on biofilm formation and filamentation. (A) Wild-type and P*_TDH3_-RME1* strains in the SC5314 and L26 backgrounds were assayed for biofilm formation ability in RPMI + 10% FBS at 37°C for 24 hours in 96-well plates. Representative apical views are shown. The white scale bars indicate 100 µm. (**B**) Representative side views are shown for the panel **A** samples. (**C**) Biofilm volume was measured for biological triplicates of wild-type and P*_TDH3_-RME1* strains. (**D**) Wild-type and P*_TDH3_-RME1* strains were assayed for hypha formation ability in RPMI at 37°C for 4 hours (planktonic conditions). This medium yielded a clearer phenotypic difference than RPMI + 10% FBS (Fig. S2). The white scale bars indicate 50 µm. (**E**) Cell lengths were quantified for the panel **D** samples. At least 4 fields of view and 100 cells were examined. Statistical analysis was conducted using one-way ANOVA, and asterisks denote statistically significant differences. ***P value < 0.001 and ****P value < 0.0001.

Because several genes associated with adhesion and biofilm formation were repressed by P*_TDH3_-RME1*, we hypothesized that *RME1* may be a biofilm inhibitor. Indeed, under strong biofilm-promoting conditions, SC5314 and L26 produced substantial biofilms whereas their P*_TDH3_-RME1* derivatives did not (Fig. 2A, B and C). Defects were evident in both apical and side views; biofilm volumes were reduced roughly 10-fold (Fig. 2C). Similar results were obtained with three additional strains (Fig. S1). P*_TDH3_-RME1* reduced hypha formation as well (Fig. 2D and E; Fig. S2). These results indicate that *RME1* overexpression inhibits biofilm and hypha formation.

### Impact of loss of *RME1* function on biofilm formation

If Rme1 is a biofilm inhibitor, we expect an *rme1*Δ/Δ mutation to improve biofilm formation. However, *rme1*Δ/Δ mutations did not alter biofilm production or hypha formation (Fig. S3). The data of Mundodi et al. indicate that *RME1* RNA levels are downregulated in hyphae compared with yeast cells (31). Therefore, we considered the possibility that *RME1* is naturally repressed during biofilm and hypha formation.

*RME1* overexpression inhibits biofilm formation in diverse strains, so it stands to reason that a repressor of *RME1* must be active in diverse strains as well. Efg1 and Brg1 are candidate *RME1* repressors because they are required for biofilm formation in diverse strains (30, 32). This idea predicts that *rme1*Δ/Δ mutations may restore biofilm and hypha formation in *efg1*Δ/Δ or *brg1*Δ/Δ mutants.

**Fig 3:**
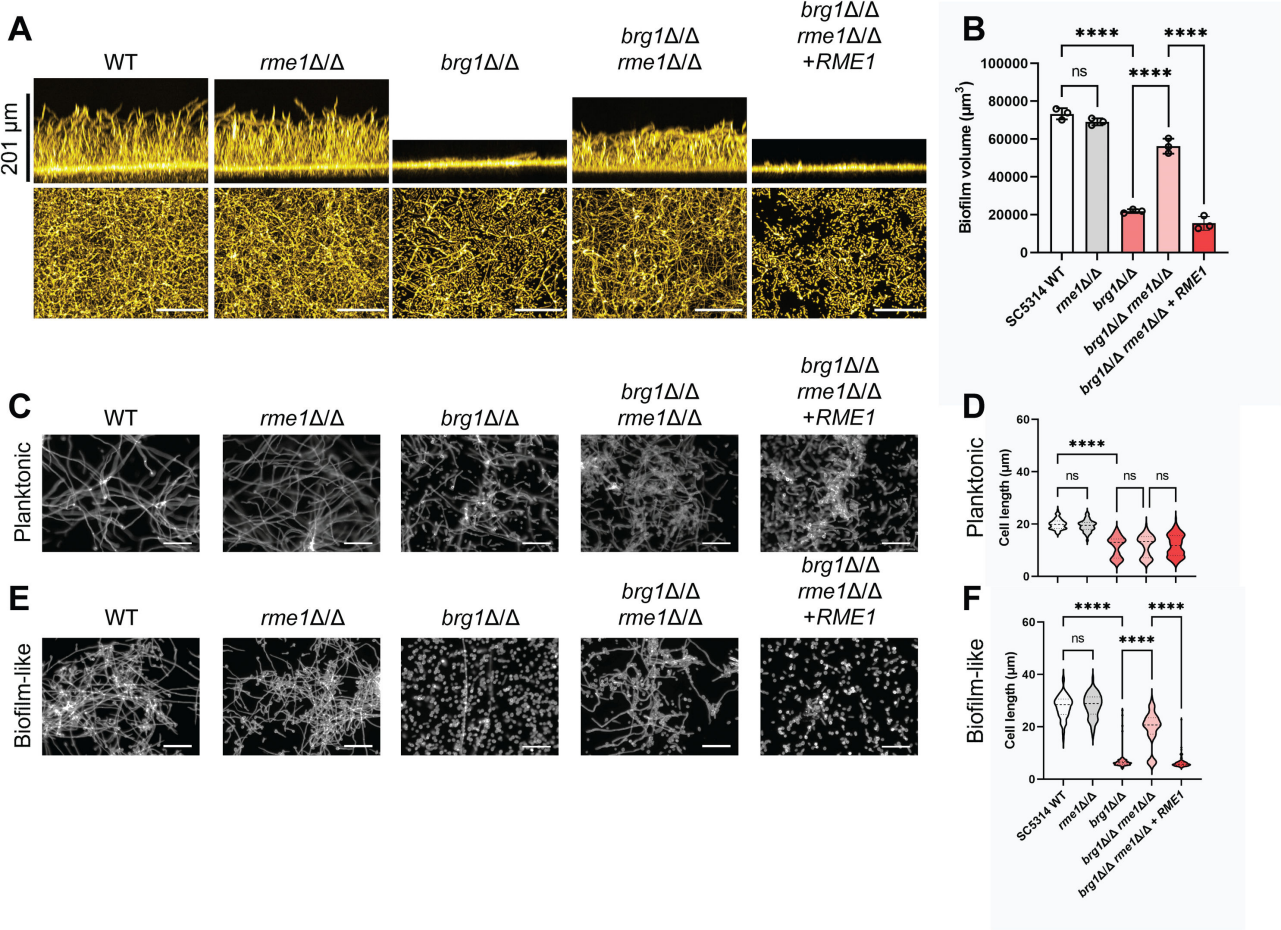
Impact of *rme1*Δ/Δ on biofilm formation and filamentation. (A) Biofilm assays were conducted on the wild type, *rme1*Δ/Δ and *brg1*Δ/Δ single mutants, a *brg1*Δ/Δ *rme1*Δ/Δ double mutant, and a *brg1*Δ/Δ *rme1*Δ/Δ + *RME1* complemented strain in the SC5314 reference background. Biofilm formation was assayed in RPMI + 10% FBS at 37°C for 24 hours in 96-well plates. Representative side (above) and apical (below) views are shown. The white scale bars indicate 100 µm. (**B**) Biofilm volume was measured for biological triplicates of the panel **A** strains. (**C**) Filamentation was assayed for the indicated strains in planktonic conditions: RPMI medium, 30 hours, 37°C with vigorous shaking. Representative images are shown. The white scale bars indicate 50 µm in length. (**D**) Cell length was measured for the panel **C** strains. (**E**) Filamentation was assayed for the indicated strains in biofilm-like conditions: RPMI medium, 30 hours, 37°C with sealed lids and no shaking. Representative images are shown. The white scale bars indicate 50 µm in length. (**F)** Cell length was measured for the panel **E** strains. For measurements in panels **D** and **E**, at least 4 fields of view and 100 cells were examined. Statistical analysis for panels **B**, **(D**, and **E** was conducted using a one-way ANOVA, and asterisks denote statistically significant differences. ****P value < 0.0001.

We tested the prediction with biofilm assays of *efg1*Δ/Δ *rme1*Δ/Δ and *brg1*Δ/Δ *rme1*Δ/Δ double mutants in the SC5314 reference background. There was no impact of an *rme1*Δ/Δ mutation on the *efg1*Δ/Δ biofilm defect (Fig. S4); both *efg1*Δ/Δ and *efg1*Δ/Δ *rme1*Δ/Δ strains were biofilm defective compared with the wild type. In contrast, an *rme1*Δ/Δ mutation substantially improved biofilm formation in a *brg1*Δ/Δ mutant background; the *brg1*Δ/Δ *rme1*Δ/Δ double mutant produced biofilm depth and volume approaching that of the wild type (Fig. 3A and B). Introduction of wild-type *RME1* reversed the effect of the *rme1*Δ/Δ mutation and restored a biofilm defect (Fig. 3A and B). These results show that *RME1* expression contributes to the *brg1*Δ/Δ biofilm defect and are consistent with the hypothesis that Brg1 represses *RME1*.

### Impact of loss of *RME1* function on filamentation

Filamentation is critical for biofilm formation (33) and depends upon Brg1 (27, 28). To see if Rme1 mediates Brg1 control of filamentation, we compared relevant strains in the SC5314 background. We began with conventional planktonic filamentation tests, in which cells were incubated under strongly inducing conditions (RPMI + 10% FBS, 37°C) for 30 hours with vigorous aeration (Fig. 3C and D; Data set S1). The wild type and *rme1*Δ/Δ mutant produced similar levels of filamentation; the *brg1*Δ/Δ mutant was defective, as expected. Surprisingly, the *brg1*Δ/Δ *rme1*Δ/Δ double mutant presented a filamentation defect similar to the *brg1*Δ/Δ single mutant. Comparable results were obtained with a shorter 4-hour incubation (Fig. S5). These results seemed inconsistent with biofilm assays of the strains presented above.

We then conducted filamentation tests under biofilm-like conditions (34). For these assays, cultures were grown in capped tubes to limit exchange of gasses and were incubated statically to allow local accumulation of secreted metabolites and quorum-sensing molecules. Assays were conducted at 37°C in RPMI + 10% FBS, just as for the planktonic filamentation assays above (Fig. 3E and F; Data Set S1). *RME1* RNA levels were strongly induced under biofilm-like conditions compared with planktonic conditions (Fig. S6). Again, the wild type and *rme1*Δ/Δ mutant produced similar levels of filamentation; the *brg1*Δ/Δ mutant was defective. However, the *brg1*Δ/Δ *rme1*Δ/Δ double mutant presented much higher levels of filamentation than the *brg1*Δ/Δ single mutant. *RME1* complementation in the *brg1*Δ/Δ *rme1*Δ/Δ + *RME1* strain restored a filamentation defect. These results show that *RME1* expression contributes to the *brg1*Δ/Δ filamentation defect as well as its biofilm defect and indicate that the activity of Rme1 is dependent upon growth in biofilm-like conditions.

### RNA-seq analysis of the Brg1-Rme1 circuit

To investigate the gene expression impact of Brg1 and Rme1, we conducted RNA-seq analysis. Cells were grown under the same biofilm-like conditions (RPMI + 10% FBS, 37°C, static incubation, 30 hours) used for filamentation assays. We used the SC5314 reference background and included wild-type, *rme1*Δ/Δ, *brg1*Δ/Δ, and *brg1*Δ/Δ *rme1*Δ/Δ strains.

**Fig 4:**
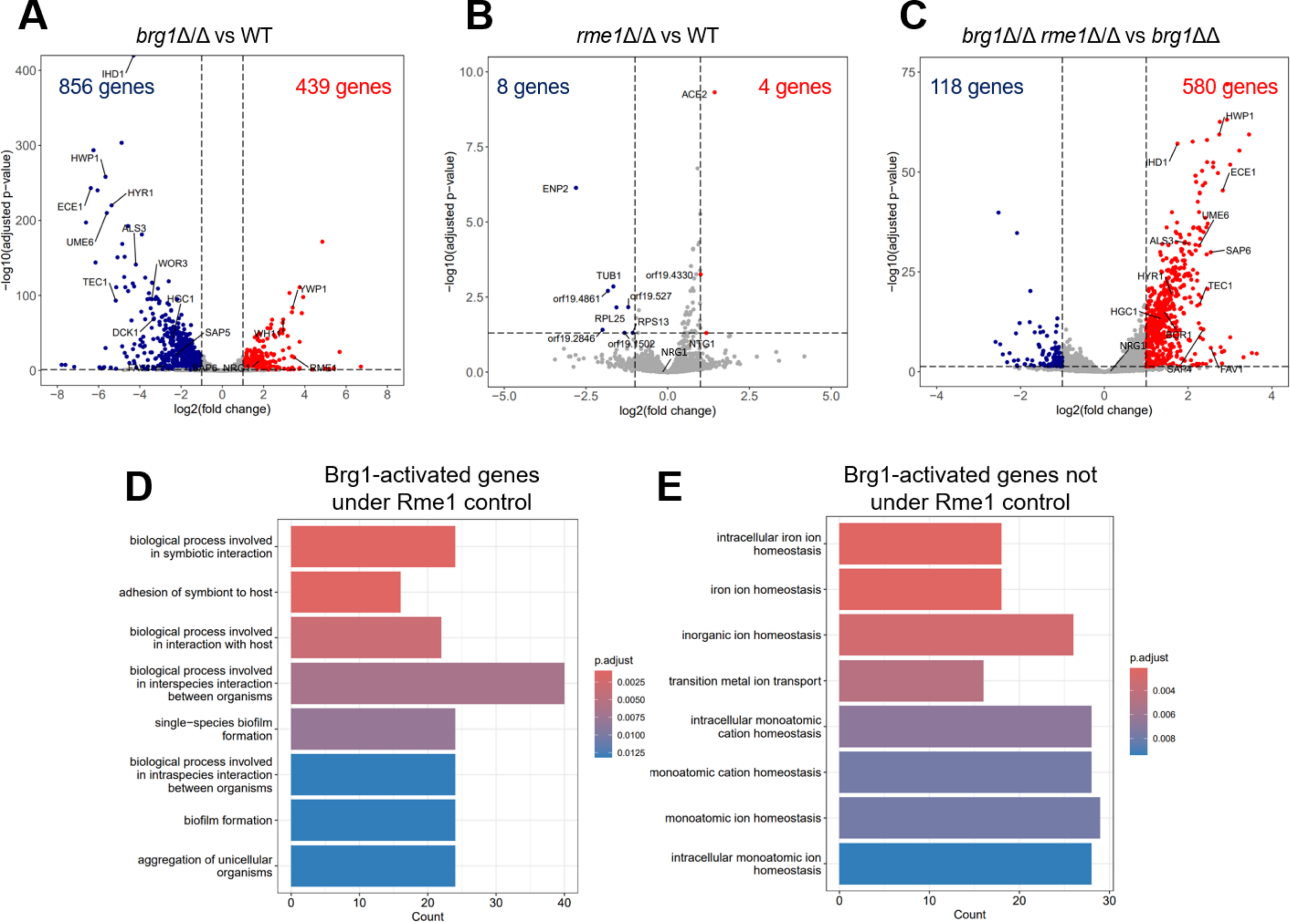
RNA-seq analysis of the Brg1-Rme1 circuit. RNA-seq analysis was conducted with cells grown under biofilm-like conditions (RPMI + 10% FBS, 37°C, static incubation, 30 hours). Wild-type, *rme1*Δ/Δ, *brg1*Δ/Δ, and *brg1*Δ/Δ *rme1*Δ/Δ strains from the SC5314 reference background were used. Numerical data are in Table S2. (**A**) Gene expression changes in the *brg1*Δ/Δ mutant vs the wild type. (**B**) Gene expression changes in the *rme1*Δ/Δ mutant vs the wild type. (**C**) Gene expression changes in the *brg1*Δ/Δ *rme1*Δ/Δ double mutant vs the *brg1*Δ/Δ single mutant. (**D**) GO term summary of the 377 genes that are downregulated in the *brg1*Δ/Δ vs wild type comparison and upregulated in the *brg1*Δ/Δ *rme1*Δ/Δ vs *brg1*Δ/Δ comparison. (**E**) GO term summary of the 479 genes that are downregulated in the *brg1*Δ/Δ vs wild type comparison and not upregulated in the *brg1*Δ/Δ *rme1*Δ/Δ vs *brg1*Δ/Δ comparison.

Brg1 is a well-established positive regulator of hypha-associated genes (27, 28). Results of our *brg1*Δ/Δ vs wild type comparison (Fig. 4A; Table S2) were consistent with expectation. Numerous hypha-associated genes (*ALS3*, *ECE1*, *HWP1*, and *UME6*) were downregulated, and yeast-associated genes (*YWP1* and *NRG1*) were upregulated, in the *brg1*Δ/Δ mutant. Importantly, *RME1* was upregulated in the *brg1*Δ/Δ mutant, as expected if Brg1 is a repressor of *RME1* expression.

Strains lacking only Rme1 have no detected biological phenotype under biofilm-like conditions. Results of our *rme1*Δ/Δ vs wild type comparison (Fig. 4B; Table S2) aligned with those observations: expression of only 12 genes was affected by the *rme1*Δ/Δ mutation. This finding is consistent with the idea that *RME1* is normally repressed under biofilm- and hyphal-inducing conditions.

Rme1 seems to be active in the absence of Brg1 under biofilm-like conditions. Results of our *brg1*Δ/Δ *rme1*Δ/Δ vs *brg1*Δ/Δ comparison (Fig. 4C; Table S2) agreed with that idea. Numerous hypha-associated genes were upregulated in the *brg1*Δ/Δ *rme1*Δ/Δ strain. Upregulation of hyphal cyclin gene *HGC1* explains how the *rme1*Δ/Δ mutation improves filamentation in the *brg1*Δ/Δ background; upregulation of adhesin genes *ALS3*, *HWP1*, and *HYR1* explains how the *rme1*Δ/Δ mutation improves biofilm formation in the *brg1*Δ/Δ background. Overall, these results align with *RME1* overexpression results to indicate that Rme1 is a negative regulator of hypha-associated genes.

The RNA-seq data above suggest that Brg1 has two functional roles that can be distinguished by the impact of Rme1. Consider the 856 genes that require Brg1 for full expression (Fig. 4A; Table S2): 377 of those genes are upregulated by loss of Rme1; 479 are not. The upregulated genes are enriched for GO terms related to biofilm formation (Fig. 4D) and include well-known hypha-associated genes. The genes that are not upregulated are enriched for GO terms related to iron homeostasis (Fig. 4E; Table S2, “iron homeostasis genes” tab) and include iron regulator *SEF1*. These results indicate that Brg1 activates hypha-associated genes through its repression of *RME1* and activates iron homeostasis genes independently of *RME1*.

Brg1 has not been recognized explicitly for its positive role in iron homeostasis gene expression to our knowledge. Detection of this effect may rely upon the biofilm-like growth conditions we employed here. However, previous ChIP-chip studies recorded Brg1 binding to iron homeostasis genes *ISU1*, *FET34*, and *FTR1* (28). In addition, Brg1 has been connected to iron homeostasis in an analysis of Irf1 (35), a transcriptional activator of iron homeostasis genes as well as *BRG1* and *EFG1* expression. It was proposed that filamentous growth may improve iron scavenging by enabling physical escape of an iron-depleted environment (35). Our data suggest that Brg1 can promote iron acquisition as well through stimulation of expression of several iron acquisition genes. Although gene activation by Brg1 of *ISU1*, *FET34*, and *FTR1* may be direct, the mechanism through which Brg1 stimulates expression of the other iron acquisition genes is uncertain.

### Context of the Rme1-Brg1 relationship

**Fig 5:**
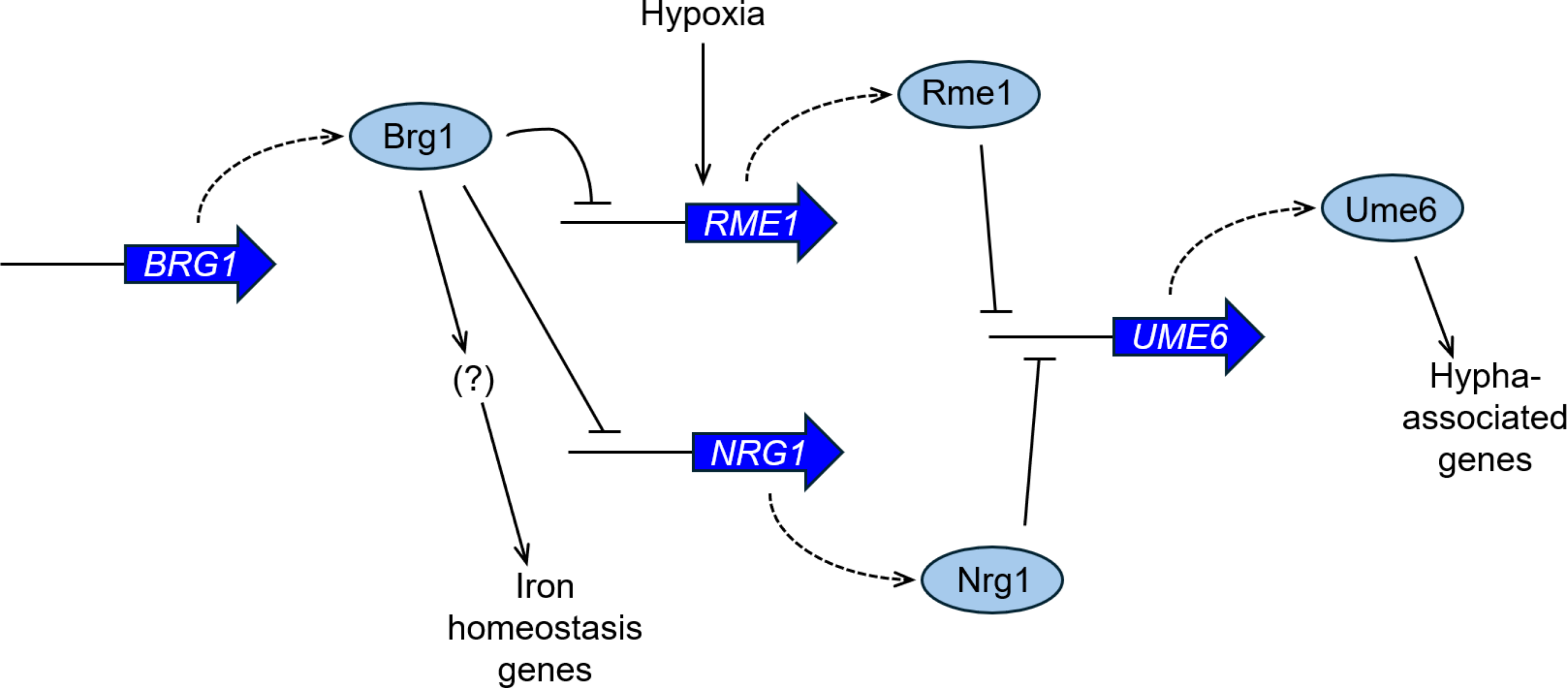
Summary of the Rme1-Brg1 relationship. Our results indicate that Rme1 acts downstream of biofilm master regulator Brg1 to control hypha-associated genes, biofilm formation, and hypha formation. We propose that Brg1 represses RME1, Rme1 represses UME6, and Ume6 activates hypha-associated genes. ChIP-chip data suggest that RME1 is repressed directly by Brg1 (28) and that *UME6* is repressed directly by Rme1 (25). Ume6 is known to be an activator of hypha-associated genes (22, 29). Our results also show that Brg1 is also required for full expression of several iron homeostasis genes and that this role is independent of Rme1. ChIP-chip data indicate that activation by Brg1 of *ISU1*, *FET34*, and *FTR1* may be direct (28) but an unidentified regulator may intercede for Brg1 to stimulate the other iron homeostasis genes. Rme1 acts in parallel with the well-established hyphal repressor Nrg1 (29), and we suggest that Rme1 and Nrg1 act independently, perhaps under distinct environmental conditions. *RME1* is induced by hypoxia (37) or, as shown here, under biofilm-like growth conditions, and *RME1* expression depends upon the hypoxia regulator Upc2 (38). The other known function of Rme1—activation of chlamydospore formation (25)—also occurs under hypoxic conditions. Thus, the natural function of Rme1 may be exerted mainly during hypoxic growth.

Two observations argue that Rme1 acts downstream of Brg1 to control hypha-associated genes (Fig. 5): *RME1* RNA levels are upregulated in a *brg1*Δ/Δ mutant, and an *rme1*Δ/Δ mutation increases hypha-associated gene expression, biofilm formation, and hypha formation in a *brg1*Δ/Δ mutant. *RME1* may be repressed directly by Brg1 because ChIP-chip data indicate that Brg1 binds to the *RME1* 5′ region (28). We hypothesize that Rme1 represses hypha-associated gene expression by repressing *UME6*, given that an *rme1*Δ/Δ mutation increases *UME6* RNA levels in a *brg1*Δ/Δ background. Repression of *UME6* may be direct, because ChIP-seq data indicate that Rme1 binds to the *UME6* 5′ region (25), though we note that binding of Rme1 occurs upstream of the 6-kbp 5′ region that is sufficient for *UME6* expression (36). Rme1 does not bind to most hypha-associated genes (25), and Ume6 is an activator of hypha-associated genes (22, 29). A simple view of this circuit is that Brg1 represses *RME1*, Rme1 represses *UME6*, and Ume6 activates hypha-associated genes.

The Brg1-Rme1 relationship parallels the Brg1-Nrg1 relationship (Fig. 5). Nrg1 is a repressor of *UME6* and hypha-associated gene expression, and it is in turn repressed by Brg1 (22, 29). *NRG1* is not regulated by Rme1 at the RNA level, based on our RNA-seq data for *rme1*Δ/Δ and *brg1*Δ/Δ *rme1*Δ/Δ mutants. *RME1* RNA levels are upregulated slightly by an *nrg1*Δ/Δ mutation in two strain backgrounds (39), a direction of change that is opposite expectation if Nrg1 represses hypha-associated genes through activation of *RME1*. These results are consistent with the idea that Rme1 and Nrg1 act independently to repress hypha-associated genes, perhaps under distinct conditions. For example, numerous studies show that Nrg1 is active under planktonic growth conditions (29, 40, 41) and our results indicate that Rme1 is not. On the other hand, Rme1 is active under biofilm growth conditions, in which an *rme1*Δ/Δ mutation is sufficient for hypha and biofilm formation in a *brg1*Δ/Δ mutant.

What restricts Rme1 activity to biofilm growth conditions? Hypoxia may be a key factor. Sellam et al. showed that *RME1* is induced rapidly when cells encounter hypoxic conditions (37). Synnott and colleagues found that *RME1* expression depends upon Upc2, a central regulator of the hypoxic gene expression response (38). The other known function of Rme1—activation of chlamydospore formation (25)—also occurs under hypoxic conditions. Where we detect the functional impact of Rme1 under ambient oxygen conditions, it depends upon *RME1* overexpression. Thus, the natural function of Rme1 may be exerted mainly during hypoxic growth.

## MATERIALS AND METHODS

### Strains and media

Clinical isolates were described previously (12, 30). Strains were frozen in 15% glycerol solution at −80°C for long-term storage. Before all experiments, strains were grown on YPD (1% yeast extract, 2% Bacto peptone, and 2% dextrose) solid medium (2% Bacto agar) at 30°C for 48 hours and cultured overnight in YPD liquid medium in a tissue culture rotator at 30°C with agitation. Transformations followed the transient CRISPR method (42). Transformant colonies were selected on CSM-His solid medium (1.7% Difco yeast nitrogen base with ammonium sulfate with amino acid supplement lacking histidine, 2% dextrose, and 2% Bacto agar), YPD + 400 µg/mL nourseothricin (clonNAT; Gold Biotechnology) solid medium, or YPD + 2 mg/mL kanamycin (G-418; Gold Biotechnology) solid medium as appropriate. Liquid RPMI-1640 medium (Sigma-Aldrich Inc., St. Louis) adjusted to pH 7.4 and supplemented with or without 10% FBS (Atlanta Biologicals Inc., Flower Branch) was used for biofilm formation, filamentation, and RNA cell cultures in both planktonic and biofilm-like conditions. All strains used in this study and their genotypes, as well as primers and plasmids, are listed in Table S3.

P*_TDH3_-RME1* overexpression strains were generated in the SC5314 and L26 wild type using a PCR primer set of RME1pro OE TDH3-NAT1/F–RME1pro OE TDH3-NAT1/R. This was done by targeting the *RME1* native promoter with a sgRNA specific to that locus; 500 bp immediately 5′ of the *RME1* was then replaced via homology-directed repair with a P*_TDH3_-RME1* construct PCR amplified from pCJN542 (43) containing 80 bp of flanking homology to the *RME1* promoter region. Resultant mutant strain genotypes were *RME1*proΔ::*NAT1-TDH3*pro. All genotypes were verified via PCR amplification of the inserted construct and native locus from genomic DNA of transformant colonies.

Homozygous knockout mutants were made using existing *his1*Δ::r1*NAT1*r1 auxotrophic SC5314 and L26 derivative strains (30). sgRNAs were made to target *ECE1* and *RME1* (referred to collectively as YFG1) in this study. Each YFG1 was replaced via homology-directed repair using a PCR-amplified recyclable *HIS1* marker from pMH01 and pMH02 with flanking repeats and 80 bp of homology to the target locus (44). Resultant mutant strain genotypes were *yfg1*Δ::r1*HIS1*r1.

Homozygous *ece1*Δ/Δ strains were generated in SC5314 and L26 *his1*Δ::r3*NAT1*r3 strains by integrating the *C.d.HIS1* marker at the *ECE1* locus using a primer set of ECE1 Del rHIS1r Sapl/F–ECE1 Del rHis1r Kpnl/R with amplified Cas9. The constructions were verified using two primer sets: ECE1 chk up/F–ECE1 int/R and ECE1 chk up/F–HIS1 CRIME/R. Resultant SC5314 *ece1*Δ/Δ (MC177) and L26 *ece1*Δ/Δ (MC207) mutants were constructed.

Homozygous *rme1*Δ/Δ strains were generated in SC5314, P76067, P57055, P87, P75010, P78042, and P78048 *his1*Δ::r3*NAT1*r3 strains by integrating the *C.d.HIS1* marker at the *RME1* locus using a primer set of RME1 Del rHIS1r Sapl/F–RME1 Del rHis1r Kpnl/R with amplified Cas9. The constructions were verified using two primer sets: RME1 chk up/F–RME1 int/R and RME1 chk up/F–HIS1 CRIME/R. Resultant SC5314 *rme1*Δ/Δ (MC347), P76067 *rme1*Δ/Δ (MC342), P57055 *rme1*Δ/Δ (MC345), P87 *rme1*Δ/Δ (MK971), P75010 *rme1*Δ/Δ (MK978), P78042 *rme1*Δ/Δ (MC316), and P78048 *rme1*Δ/Δ (MC339) mutants were constructed.

Homozygous *brg1*Δ/Δ *rme1*Δ/Δ strains were generated in the SC5314, P76067, P57055, P87, and P75010 *brg1*Δ/Δ strain backgrounds. The *brg1*Δ/Δ mutants from five clinical isolates became sensitive to nourseothricin by recycling the *NAT1* marker at the *his1*Δ/Δ locus when *BRG1* is deleted. The *NAT1* marker was amplified from plasmid pNAT with 80 bp of flanking homology from the up- and downstream of the *RME1* ORF region being deleted using a primer set of RME1 Del Nat1/F–RME1 Del Nat1/R. Transformant genotypes were verified using two primer sets: RME1 chk up/F–NAT1 chk int/R and RME1 chk up/F–RME1 chk int/R. SC5314 *brg1*Δ/Δ *rme1*Δ/Δ (MK939), P76067 *brg1*Δ/Δ *rme1*Δ/Δ (MK955), P57055 *brg1*Δ/Δ *rme1*Δ/Δ (MK957), P87 *brg1*Δ/Δ *rme1*Δ/Δ (MK958), and P75010 *brg1*Δ/Δ *rme1*Δ/Δ (MK961) mutants were constructed.

Complementation was achieved by using a native *RME1* promoter-RME1 ORF PCR construct using two primer sets: RME1 5′/F–RME1 3′ → CaKan 5′/R and CaKan adapt/F–CaKan 3 → RME1 adapt/R″. For CaKan amplification, the pSFS2A-CaKan plasmid was used (45), obtained from Addgene (plasmid # 189565). This was transformed into the SC5314 *brg1*Δ/Δ *rme1*Δ/Δ mutant strain (MK939) at the native locus of *RME1*, and the constructions were verified using three primer sets: RME1 far chk up/F–RME1 int/R, RME1 far chk up/F–CaKan chk int/R, and RME1 far chk up/F–NAT1 int/R. The *rme1*Δ::*RME1*-Kanamycin were reconstituted in SC5314 *brg1*Δ/Δ *rme1*Δ/Δ mutant, constructing SC5314 *brg1*Δ/Δ *rme1*Δ/Δ + *RME1* mutant (MK987).

### Gene co-expression clustering analysis

To identify candidate genes driving epithelial cell damage ability, we utilized co-expression clustering to identify genes whose expression levels in wild-type (WT) isolates correlate with epithelial cell damage. We used RNA-seq data from Cravener et al. (30) for 17 WT clinical isolates, cultured in RPMI + 10% FBS at 37°C for 4 hours with vigorous shaking before RNA extraction and sequencing. As described (30), counts were normalized using DESeq2 and a median expression level for each gene across all strains was calculated. Log_2_ fold change expression levels were then calculated relative to the 17-strain median for each strain. For our analysis here, genes were filtered for those with more than 200 normalized counts in one or more samples and magnitude log_2_ fold change of 0.5 in at least one WT strain background, yielding 2,125 genes. Expression profiles for high-damage (SC5314 and L26) and low-damage strains (P76067, P78042, and P78048) were then clustered using a self-organizing tree algorithm (SOTA) with a maximum cell diversity of 0.9 and subsequent hierarchical clustering (Pearson correlation) of both genes and samples for each SOTA cluster. The SOTA method generated 41 total clusters; two clusters were positively correlated with epithelial cell damage; three clusters were anticorrelated. In total, we found 507 genes that correlated or anticorrelated with epithelial cell damage (Table S1).

### Epithelial cell damage

The extent of damage to the oral epithelial cells caused by different *C. albicans* strains was measured by a ^51^Cr release assay in 96-well plates as described previously (21). The inoculum was 2.5 × 10^5^ cells per well, and the incubation period was 6 hours. Each experiment was repeated three times in triplicate.

### Biofilm formation assays

Biofilm formation assays were conducted in 96-well plates (Greiner 96-well plate; Cat #. 655090) with the protocol described by Do et al. (19). Strains were cultured overnight in YPD liquid medium. Strains were then inoculated to a final optical density at 600 nm (OD_600_) of 0.05 in 100 µL of pre-warmed RPMI or RPMI + 10% FBS medium and incubated at 37°C for 90 minutes in a shaking shaker with 60 rpm to allow adherence. Afterwards, wells were gently washed twice with phosphate-buffered saline (PBS) to remove non-adherent cells and 100 µL of pre-warmed and fresh RPMI or RPMI + 10% FBS medium was added to each well. Cells in the 96-well plate were incubated at 37°C with 60 rpm agitation for 24 hours. Next, the medium was removed, and biofilms were fixed during incubation with 100 µL of 4% formaldehyde in PBS solution at room temperature for 1 hour. Biofilms were washed twice with PBS solution and stained using 200 ng/mL of calcofluor-white in PBS solution overnight at room temperature with 60 rpm shaking. The biofilms were washed with PBS twice. Thiodiethanol (TDE) was then used to clarify biofilms: we added 100 µL of 50% TDE in PBS and allowed the plate to incubate for 1 hour at room temperature. After the solution was removed from each well, we added 100% TDE into the plate and allowed an additional 1-hour incubation at room temperature. Biofilm in each well was then imaged on Keyence BZ-X800E fluorescence microscope using 20× with a 2× digital zoom.

### Biofilm image processing

Apical and side view projections of biofilms were observed from Z-stack images as described (19, 30, 46). The Z-stacks were combined and processed using the FIJI software program (ImageJ v1.53) (47). First, Z-stacks were converted to 32-bit from 8-bit and the background signal was subtracted using the background subtract plugin. To obtain side-view images, Z-stacks reslicing and subsequent maximum intensity projection were conducted. Next, the side-view images were rescaled based on the objective used for Keyence-derived images. Apical view projections of the biofilms were created using maximum-intensity Z-projection. For both side and apical view images, brightness was adjusted and coloration to yellow was achieved.

### RNA extraction and sequencing

Strains were cultured for RNA extraction essentially as described previously (19, 30). Wild-type and mutant strains were grown overnight in 5 mL YPD liquid medium in a tissue culture rotator at 30°C. For RNA extraction from planktonic conditions, prewarmed 125-mL flasks with 25 mL RPMI or RPMI + 10% FBS were then inoculated to an OD_600_ of 0.2. Cultures were grown for 4 or 30 hours (as specified) at 37°C with shaking at 225 rpm. For RNA extraction from biofilm-like conditions, prewarmed 20-mL glass vials with 20 mL RPMI were inoculated to an OD_600_ of 0.5 and were sealed with parafilm. Cultures were grown for 4 or 30 hours (as specified) at 37°C without any agitation.

Triplicate RPMI + 10% FBS or RPMI cultures were made from the same overnight culture for each WT, P*_TDH3_-RME1*, *rme1*Δ/Δ, *brg1*Δ/Δ, and *brg1*Δ/Δ *rme1*Δ/Δ strain. Cells were obtained from vacuum filtration and quickly frozen at −80°C prior to RNA extraction. RNA extraction was employed by physically disrupting cells with Zirconia beads (Ambion, Fisher Scientific, Waltham, MA). RNA was isolated using 25:24:1 phenol:chloroform:isoamyl alcohol, followed by Qiagen RNeasy Mini Kit (Qiagen, Venlo, Netherlands) modified procedures.

For RNA-seq sample preparation, 1 µg RNA per sample was used and sequencing libraries were generated by using a NEBNext Ultra RNA Library Prep Kit for Illumina (NEB, USA). From both ends of each cDNA fragment using the Illumina platform, 150 nt of sequence was determined. Sequencing reads were aligned to the *C. albicans* reference (Assembly A21) using HISAT2. DESeq2 R package (version 1.40.2) was utilized to conduct differential expression analysis between two groups, each with three biological replicates. For conducting GO term analyses, we employed clusterProfiler (v4.8.1) in R. Specifically, we generated a GO term library utilizing FungiDB (*Candida albicans*.Eupath.v63) through the R AnnotationForge package (48, 49). Genes were defined by an adjusted *P* value of less than or equal to 0.05 and by a fold change on a log_2_ scale of greater than 1 or less than −1. GO categories only with a *P* value of less than or equal to 0.05 were considered to be significant. Volcano plots were created using the ggplot2 (v3.4.2) package.

### qRT-PCR assays

Quantitative reverse transcription-polymerase chain reaction (qRT-PCR) was examined to assess *RME1* mRNA levels in three biological replicates of SC5314 wild-type and *brg1*Δ/Δ strains grown in planktonic or biofilm-like conditions. Extracted RNA was digested with DNase I and then reverse transcribed to cDNA via the iScript cDNA synthesis kit (Bio-Rad, Hercules, CA, Cat.# 172-5034). Subsequently, qRT-PCR was conducted with iQ SYBR green Supermix (Bio-Rad, Hercules, CA, Cat.# 170-8880). *RME1* mRNA levels (primers RME1 qRT PCR/F and RME1 qRT PCR/R) were normalized to the *SPA2* gene (primers SPA2 qRT PCR/F and SPA2 qRT PCR/R) and calculated by the threshold cycle ΔΔ*C_T_* method. A one-way ANOVA test was used for analysis of differences of strains and conditions.

### Filamentation assays in planktonic and biofilm-like conditions

Assays were conducted as previously described by Huang et al. (32). Wild-type, *rme1*Δ/Δ, *brg1*Δ/Δ, *brg1*Δ/Δ *rme1*Δ/Δ, and *brg1*Δ/Δ *rme1*Δ/Δ + *RME1* strains were grown overnight in YPD liquid medium in a tissue culture rotator at 30°C. The pre-warmed 5-mL aliquots of RPMI medium were inoculated from the overnight cultures to achieve an OD_600_ of 0.5, followed by incubation at 37°C for either 4 or 30 hours, with agitation at 60 rpm or without agitation. Samples for filamentation were collected through centrifugation and then fixed in 4% formaldehyde in PBS solution for 15 minutes. Afterwards, the samples were washed in PBS twice and stained with a florescent dye, Calcofluor-white. Cell imaging was conducted utilizing a slit-scan confocal unit on a Zeiss Axiovert 200 microscope equipped with a Zeiss C-Apochromat 40×/1.2 NA water immersion objective. Hyphal induction was determined by measuring the filament unit length either from yeast cell to filament tip or between septations in ImageJ. At least 100 filament units were quantified for each strain from three separate fields of view.

### Software

Images were processed and adjusted in ImageJ (47). Sample comparisons were conducted with GraphPad Prism version 10.00 (GraphPad Software Inc., La Jolla). Genome sequences, annotations, and phenotype information were retrieved from the Candida Genome Database (50) and FungiDB (51).

## Data Availability

Strains and plasmids are available upon request. The authors affirm that all data necessary for confirming the conclusions of the article are present within the article, figures, and tables. RNA-seq reads are available through NCBI under BioProject IDs PRJNA961908 and PRJNA1078267.

## SUPPLEMENTAL MATERIAL

Dataset S1 mbio.01872-24-s0001.pdf Enlarged images from Fig. 3C and E.

Supplemental figures mbio.01872-24-s0002.pdf Figures S1 to S6.

Table S1 mbio.01872-24-s0003.xlsx Cluster analysis outcomes.

Table S2 mbio.01872-24-s0004.xlsx RNA-seq data.

Table S3 mbio.01872-24-s0005.xlsx Strains, primers, and plasmids.

ASM does not own the copyrights to Supplemental Material that may be linked to, or accessed through, an article. The authors have granted ASM a non-exclusive, world-wide license to publish the Supplemental Material files. Please contact the corresponding author directly for reuse.
