# Supplementary material for: A Brg1-Rme1 circuit in Candida albicans hyphal gene regulation
Source: mBio. 2024 Jul 30;15(9):e01872-24. doi: 10.1128/mbio.01872-24 (PMC11389389; doi:10.1128/mbio.01872-24)
Supplement: Dataset S1 — Enlarged images from Fig. 3C and E. [file mbio.01872-24-s0001.pdf]

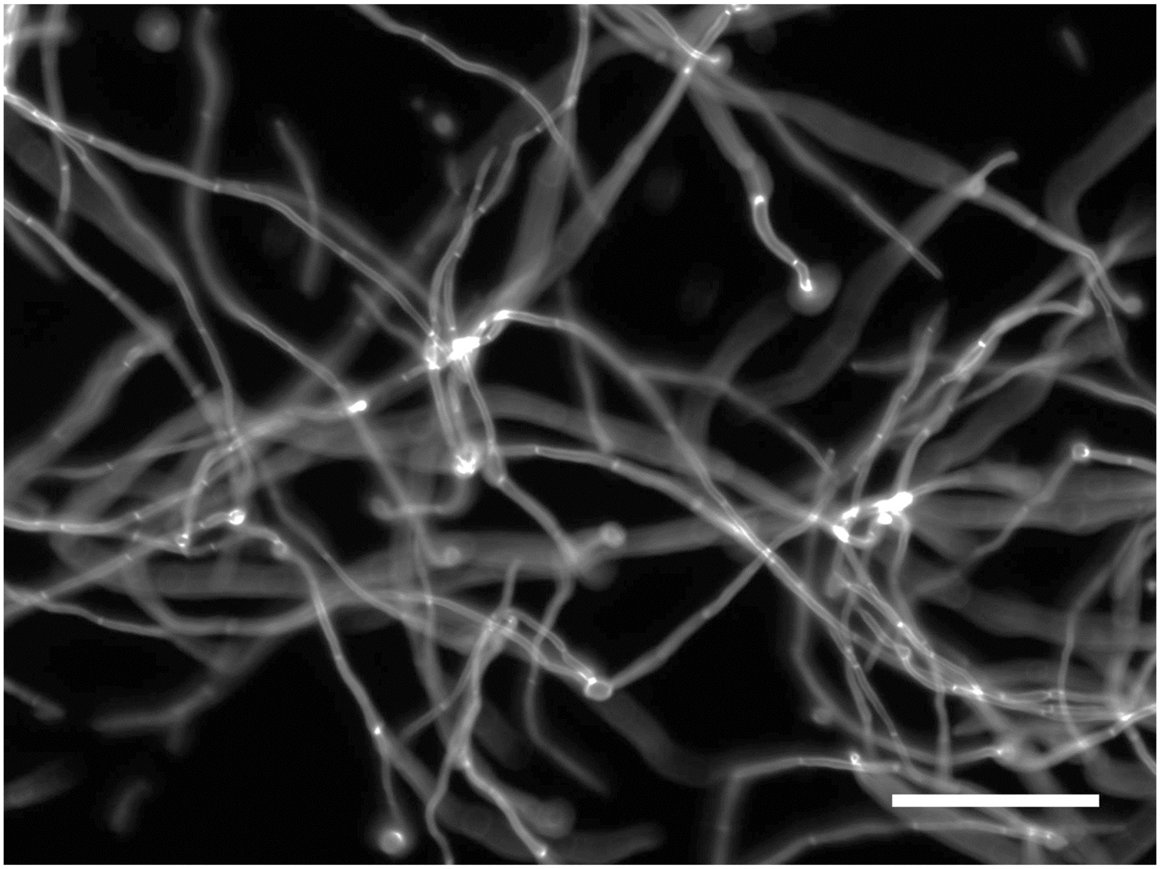

Planktonic WT

Dataset S1: Enlarged Figure 3C and E images. Impact of *rme1*Δ/Δ on biofilm formation and filamentation. Filamentation was assayed for wild type, *rme1*Δ/Δ and *brg1*Δ/Δ single mutants, a *brg1*Δ/Δ *rme1*Δ/Δ double mutant, and a *brg1*Δ/Δ *rme1*Δ/Δ+*RME1* complemented strain in the SC5314 reference background. Images are labeled ”Planktonic” (RPMI medium, 30 hours, 37°C with vigorous shaking) or “Biofilm-like” (RPMI medium, 30 hours, 37°C with sealed lids and no shaking). The white scale bars indicate 50 μm in length.

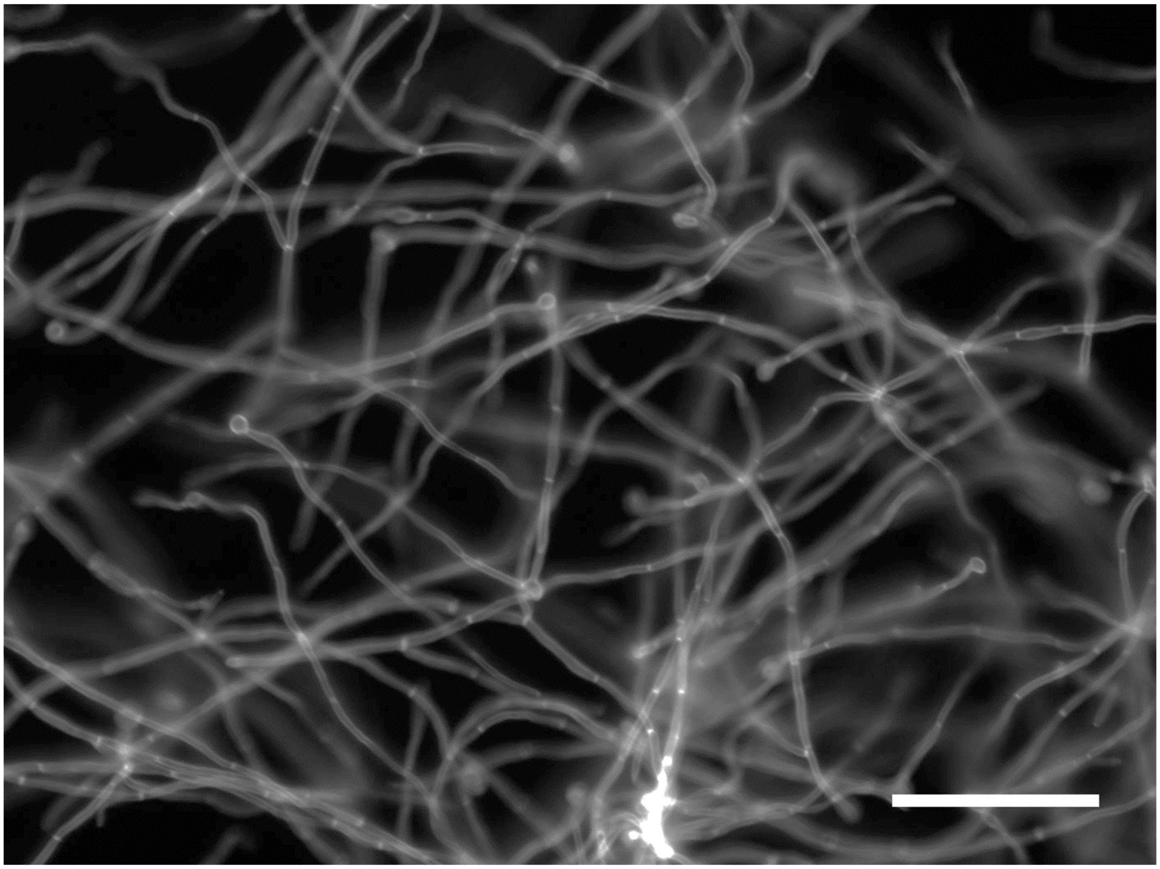

Planktonic *rme1*Δ/Δ

Dataset S1: Enlarged Figure 3C and E images. Impact of *rme1*Δ/Δ on biofilm formation and filamentation. Filamentation was assayed for wild type, *rme1*Δ/Δ and *brg1*Δ/Δ single mutants, a *brg1*Δ/Δ *rme1*Δ/Δ double mutant, and a *brg1*Δ/Δ *rme1*Δ/Δ+*RME1* complemented strain in the SC5314 reference background. Images are labeled ”Planktonic” (RPMI medium, 30 hours, 37°C with vigorous shaking) or “Biofilm-like” (RPMI medium, 30 hours, 37°C with sealed lids and no shaking). The white scale bars indicate 50 μm in length.

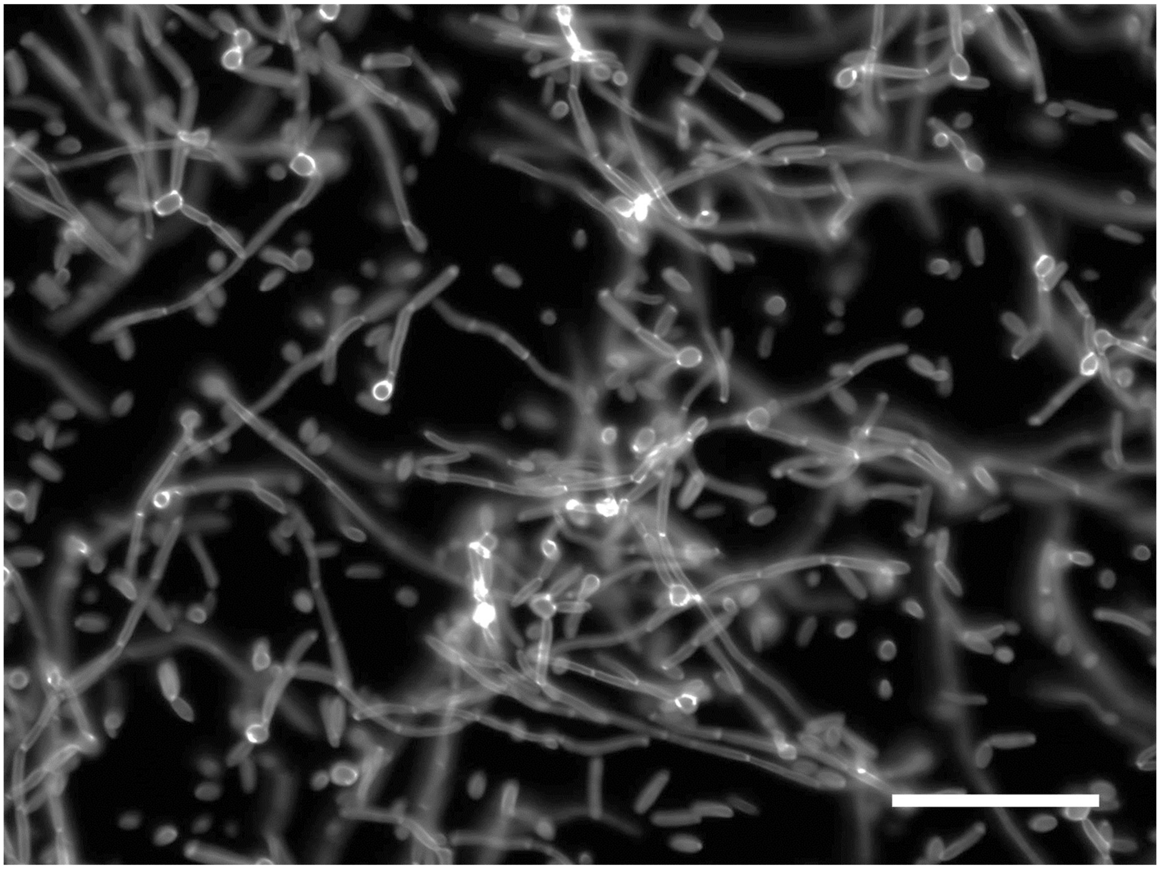

Planktonic *brg1*Δ/Δ

Dataset S1: Enlarged Figure 3C and E images. Impact of *rme1*Δ/Δ on biofilm formation and filamentation. Filamentation was assayed for wild type, *rme1*Δ/Δ and *brg1*Δ/Δ single mutants, a *brg1*Δ/Δ *rme1*Δ/Δ double mutant, and a *brg1*Δ/Δ *rme1*Δ/Δ+*RME1* complemented strain in the SC5314 reference background. Images are labeled ”Planktonic” (RPMI medium, 30 hours, 37°C with vigorous shaking) or “Biofilm-like” (RPMI medium, 30 hours, 37°C with sealed lids and no shaking). The white scale bars indicate 50 μm in length.

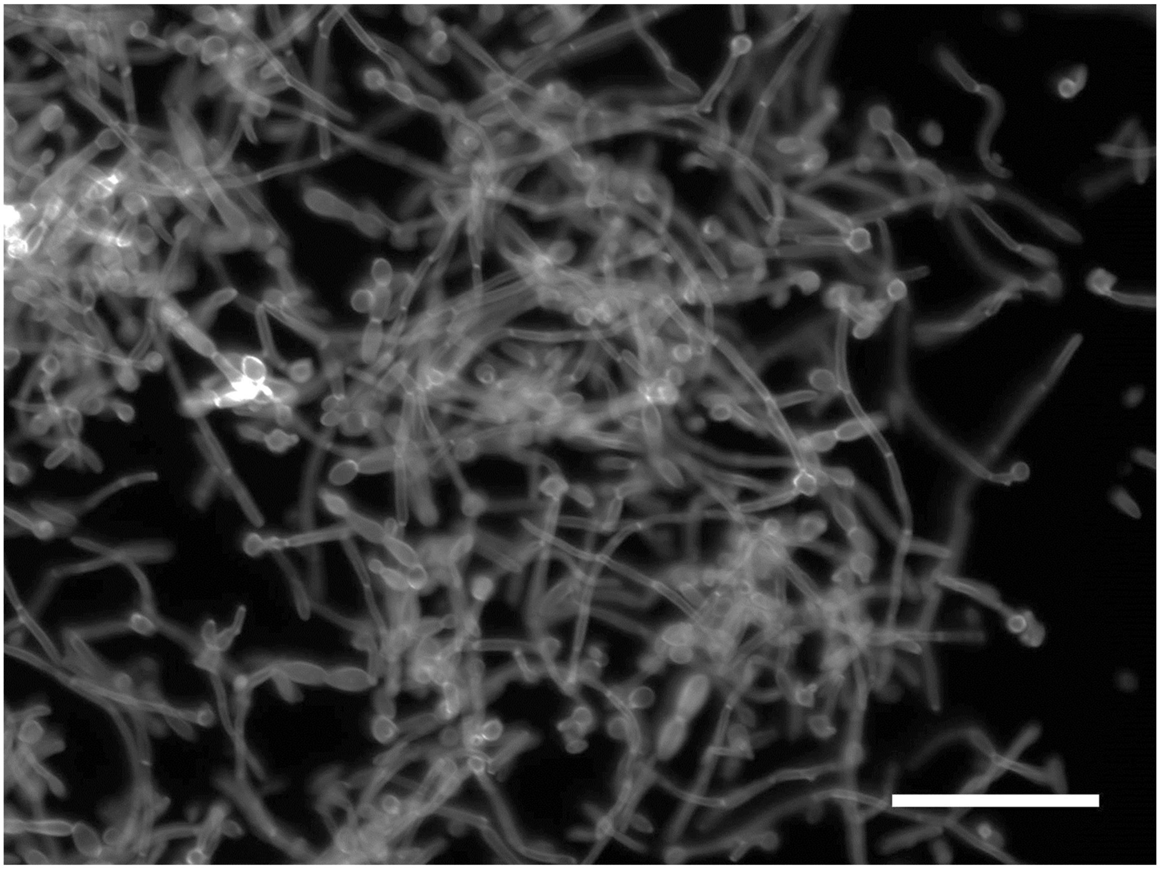

Planktonic *brg1*Δ/Δ *rme1*Δ/Δ

Dataset S1: Enlarged Figure 3C and E images. Impact of *rme1*Δ/Δ on biofilm formation and filamentation. Filamentation was assayed for wild type, *rme1*Δ/Δ and *brg1*Δ/Δ single mutants, a *brg1*Δ/Δ *rme1*Δ/Δ double mutant, and a *brg1*Δ/Δ *rme1*Δ/Δ+*RME1* complemented strain in the SC5314 reference background. Images are labeled "Planktonic" (RPMI medium, 30 hours, 37°C with vigorous shaking) or "Biofilm-like" (RPMI medium, 30 hours, 37°C with sealed lids and no shaking). The white scale bars indicate 50 μm in length.

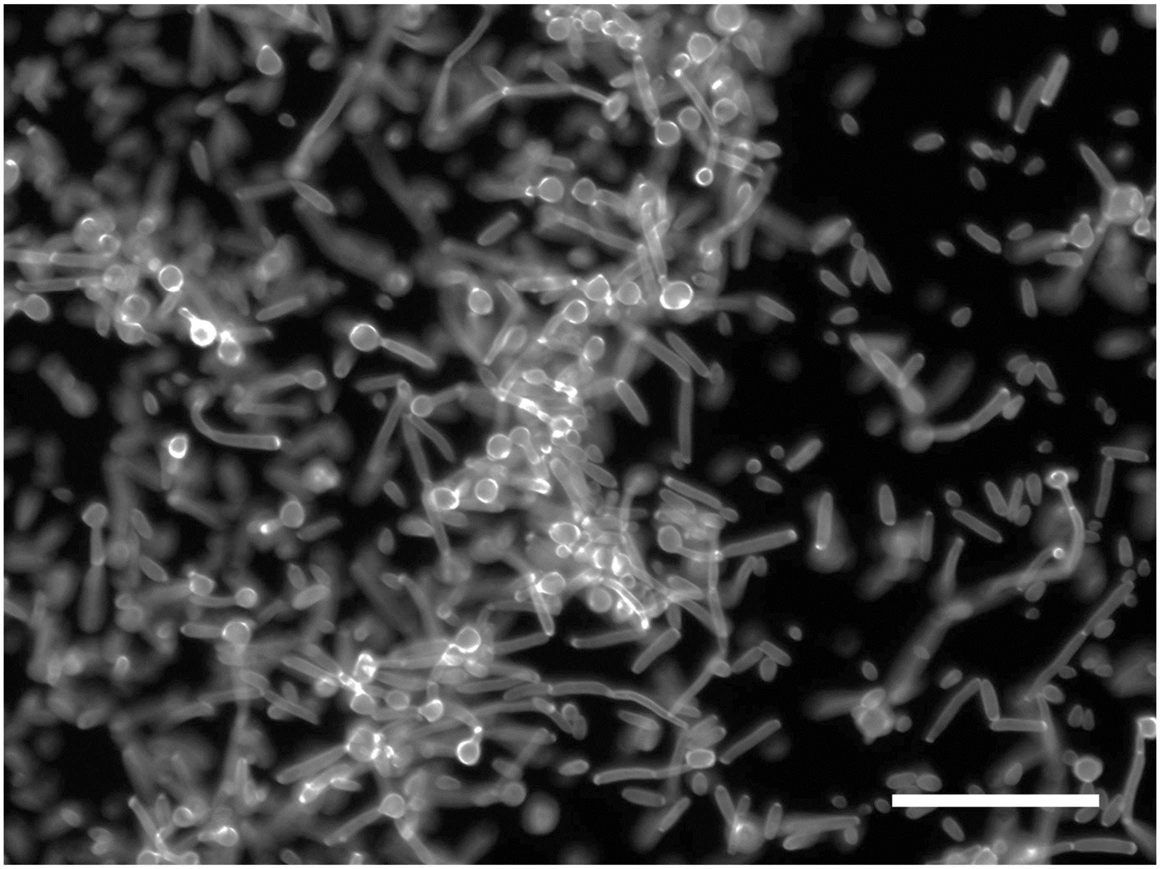

Planktonic *brg1*Δ/Δ *rme1*Δ/Δ+*RME1*

Dataset S1: Enlarged Figure 3C and E images. Impact of *rme1*Δ/Δ on biofilm formation and filamentation. Filamentation was assayed for wild type, *rme1*Δ/Δ and *brg1*Δ/Δ single mutants, a *brg1*Δ/Δ *rme1*Δ/Δ double mutant, and a *brg1*Δ/Δ *rme1*Δ/Δ+*RME1* complemented strain in the SC5314 reference background. Images are labeled "Planktonic" (RPMI medium, 30 hours, 37°C with vigorous shaking) or "Biofilm-like" (RPMI medium, 30 hours, 37°C with sealed lids and no shaking). The white scale bars indicate 50 μm in length.

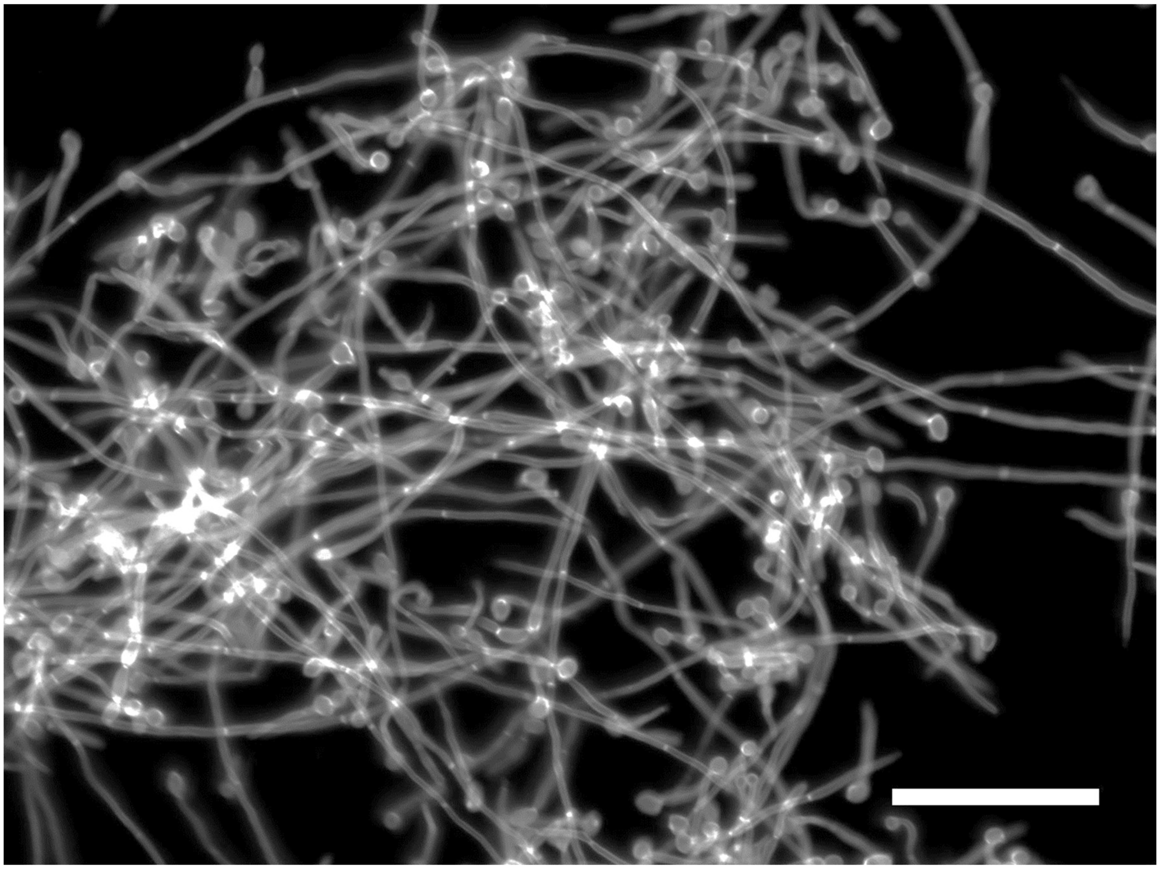

Biofilm-like WT

Dataset S1: Enlarged Figure 3C and E images. Impact of *rme1*Δ/Δ on biofilm formation and filamentation. Filamentation was assayed for wild type, *rme1*Δ/Δ and *brg1*Δ/Δ single mutants, a *brg1*Δ/Δ *rme1*Δ/Δ double mutant, and a *brg1*Δ/Δ *rme1*Δ/Δ+*RME1* complemented strain in the SC5314 reference background. Images are labeled ”Planktonic” (RPMI medium, 30 hours, 37°C with vigorous shaking) or “Biofilm-like” (RPMI medium, 30 hours, 37°C with sealed lids and no shaking). The white scale bars indicate 50 µm in length.

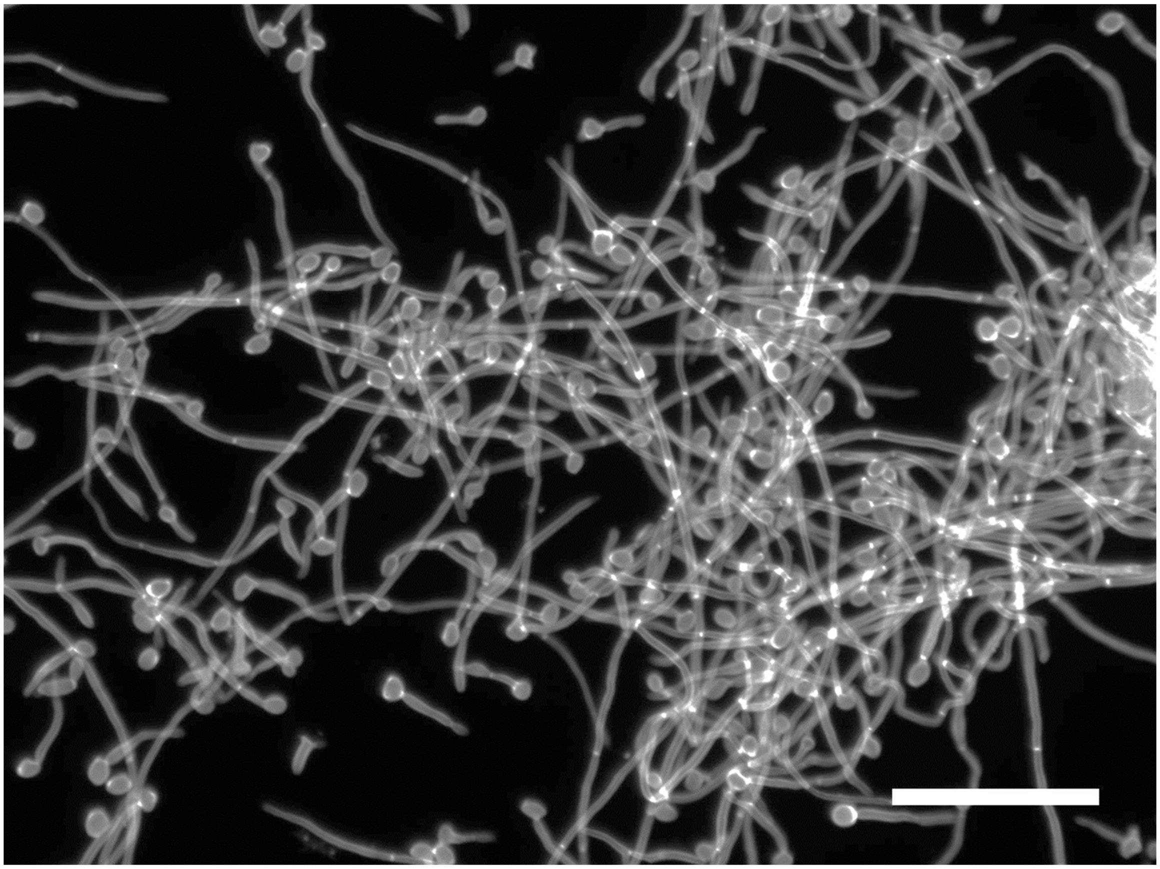

Biofilm-like *rme1*Δ/Δ

Dataset S1: Enlarged Figure 3C and E images. Impact of *rme1*Δ/Δ on biofilm formation and filamentation. Filamentation was assayed for wild type, *rme1*Δ/Δ and *brg1*Δ/Δ single mutants, a *brg1*Δ/Δ *rme1*Δ/Δ double mutant, and a *brg1*Δ/Δ *rme1*Δ/Δ+*RME1* complemented strain in the SC5314 reference background. Images are labeled "Planktonic" (RPMI medium, 30 hours, 37°C with vigorous shaking) or "Biofilm-like" (RPMI medium, 30 hours, 37°C with sealed lids and no shaking). The white scale bars indicate 50 μm in length.

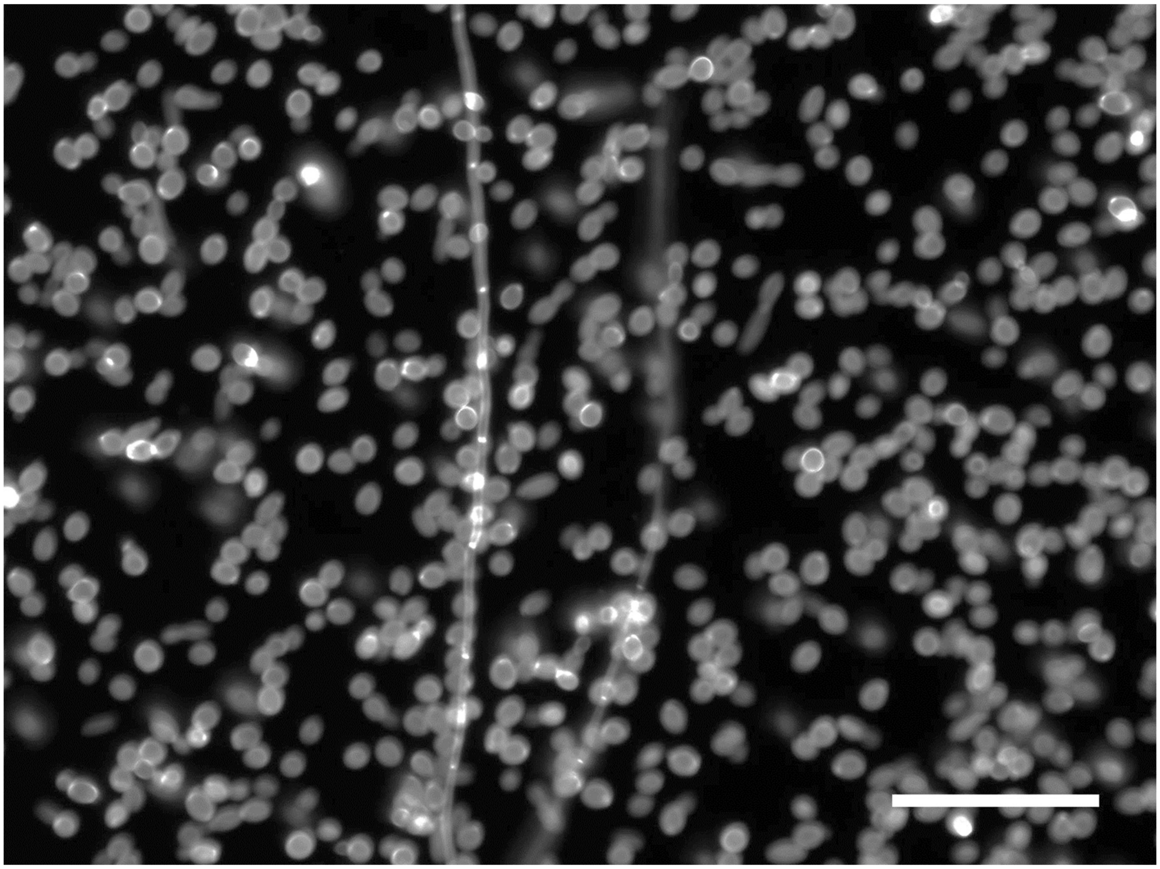

Biofilm-like *brg1*Δ/Δ

Dataset S1: Enlarged Figure 3C and E images. Impact of *rme1*Δ/Δ on biofilm formation and filamentation. Filamentation was assayed for wild type, *rme1*Δ/Δ and *brg1*Δ/Δ single mutants, a *brg1*Δ/Δ *rme1*Δ/Δ double mutant, and a *brg1*Δ/Δ *rme1*Δ/Δ+*RME1* complemented strain in the SC5314 reference background. Images are labeled "Planktonic" (RPMI medium, 30 hours, 37°C with vigorous shaking) or "Biofilm-like" (RPMI medium, 30 hours, 37°C with sealed lids and no shaking). The white scale bars indicate 50 μm in length.

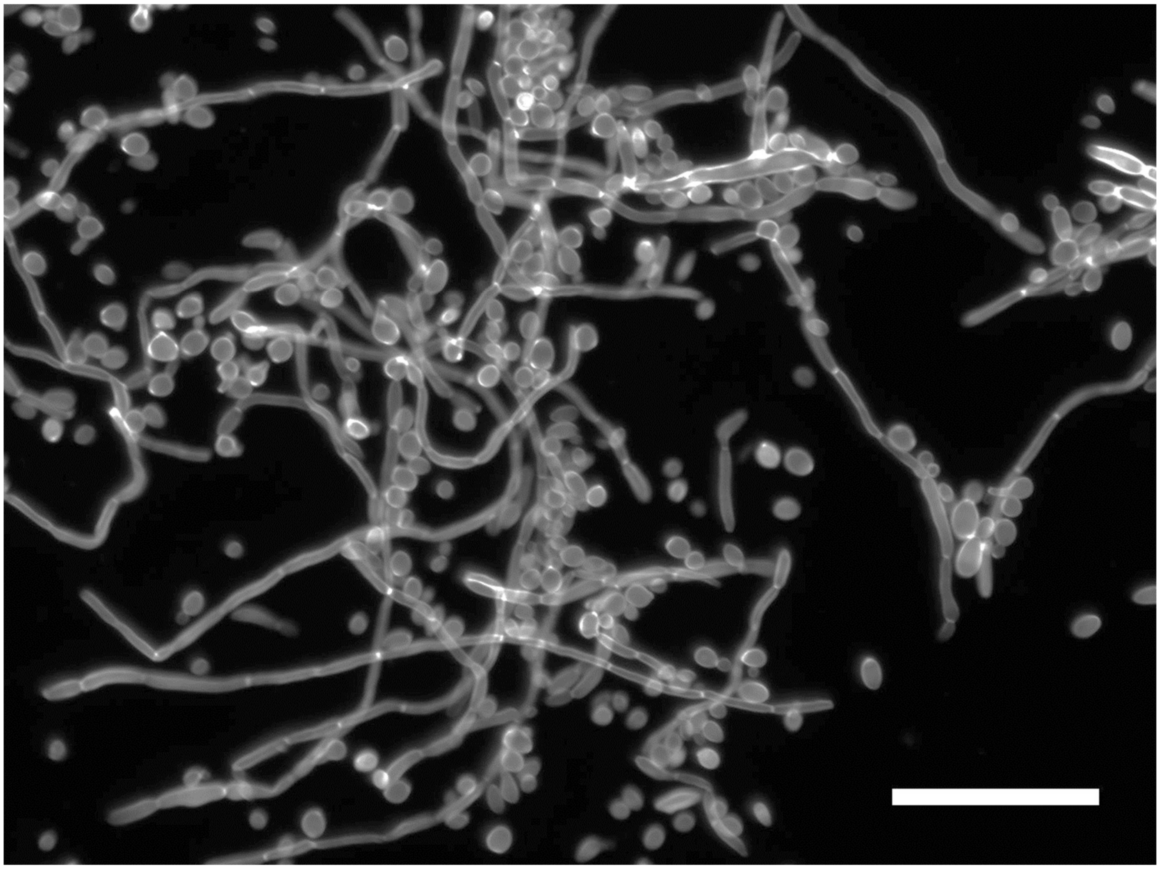

Biofilm-like *brg1*Δ/Δ *rme1*Δ/Δ

Dataset S1: Enlarged Figure 3C and E images. Impact of *rme1*Δ/Δ on biofilm formation and filamentation. Filamentation was assayed for wild type, *rme1*Δ/Δ and *brg1*Δ/Δ single mutants, a *brg1*Δ/Δ *rme1*Δ/Δ double mutant, and a *brg1*Δ/Δ *rme1*Δ/Δ+*RME1* complemented strain in the SC5314 reference background. Images are labeled "Planktonic" (RPMI medium, 30 hours, 37°C with vigorous shaking) or "Biofilm-like" (RPMI medium, 30 hours, 37°C with sealed lids and no shaking). The white scale bars indicate 50 μm in length.

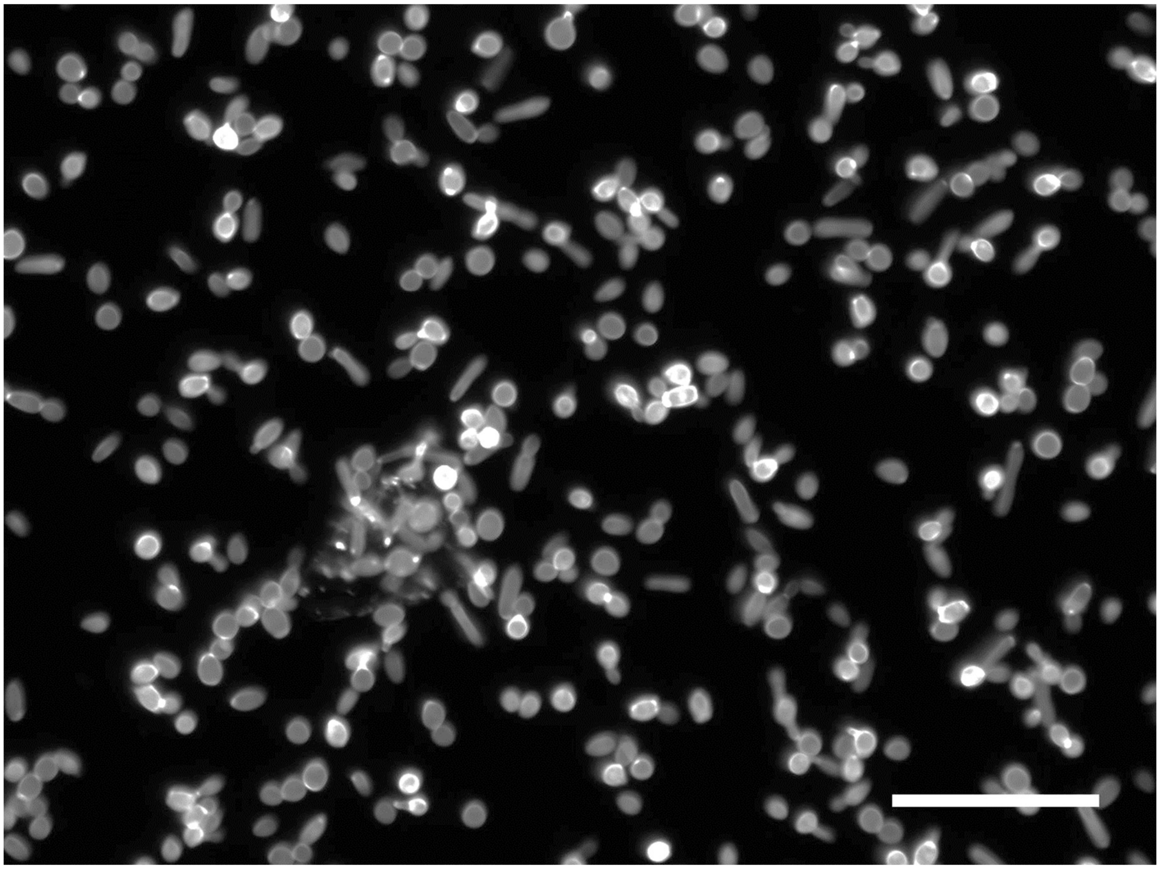

Biofilm-like *brg1*Δ/Δ *rme1*Δ/Δ+*RME1*

Dataset S1: Enlarged Figure 3C and E images. Impact of *rme1*Δ/Δ on biofilm formation and filamentation. Filamentation was assayed for wild type, *rme1*Δ/Δ and *brg1*Δ/Δ single mutants, a *brg1*Δ/Δ *rme1*Δ/Δ double mutant, and a *brg1*Δ/Δ *rme1*Δ/Δ+*RME1* complemented strain in the SC5314 reference background. Images are labeled "Planktonic" (RPMI medium, 30 hours, 37°C with vigorous shaking) or "Biofilm-like" (RPMI medium, 30 hours, 37°C with sealed lids and no shaking). The white scale bars indicate 50 μm in length.
