# Supplementary material for: A Brg1-Rme1 circuit in Candida albicans hyphal gene regulation
Source: mBio. 2024 Jul 30;15(9):e01872-24. doi: 10.1128/mbio.01872-24 (PMC11389389; doi:10.1128/mbio.01872-24)

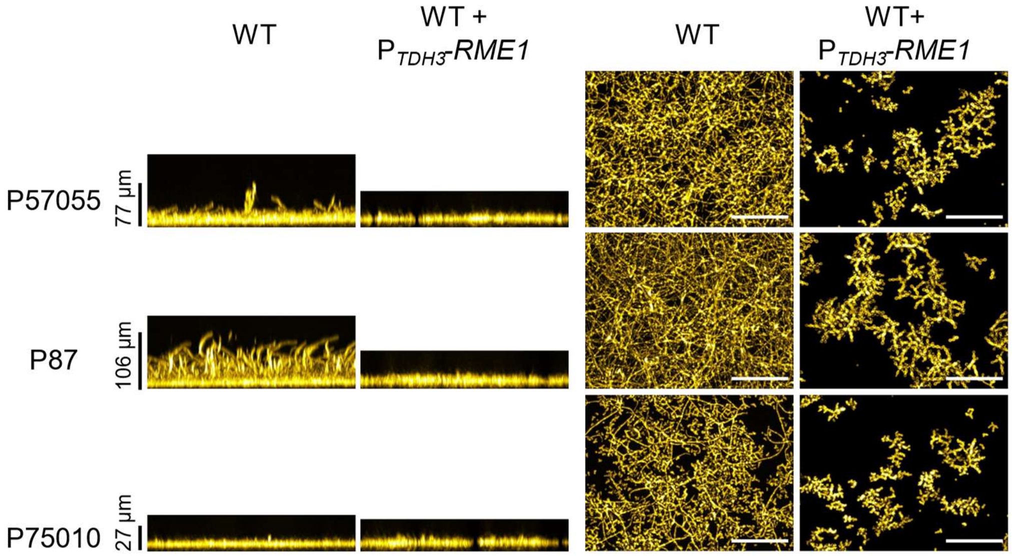

Figure S1. Impact of *RME1* overexpression on biofilm formation in additional strains. Wild-type and P_{TDH3}-*RME1* strains in the P57055, P87, and P75010 backgrounds were assayed for biofilm formation ability in RPMI at 37°C for 24 hrs in 96-well plates. Representative side (left) and apical (right) views are shown. The white scale bars indicate 100 µm.

Figure S2. Impact of *RME1* overexpression on filamentation under varied growth conditions. Wild-type and P_{TDH3}-*RME1* strains in the SC5314 and L26 backgrounds were assayed for hypha formation ability in RPMI+10% FBS (**A**) or RPMI (**B**) at 37°C for 4 hrs. Representative views are shown. The white scale bars indicate 50 µm. In each growth condition, results of planktonic assays (upper rows; grown in open tubes and shaking) or biofilm-like assays (lower rows; grown in sealed tubes and no shaking) are shown. Cell lengths were quantified (**C**) from least 4 fields of view and 100 cells. Statistical analysis was conducted using one-way ANOVA, and asterisks denote statistically significant differences. ** p-value < 0.01, *** p-value < 0.001, **** p-value < 0.0001.

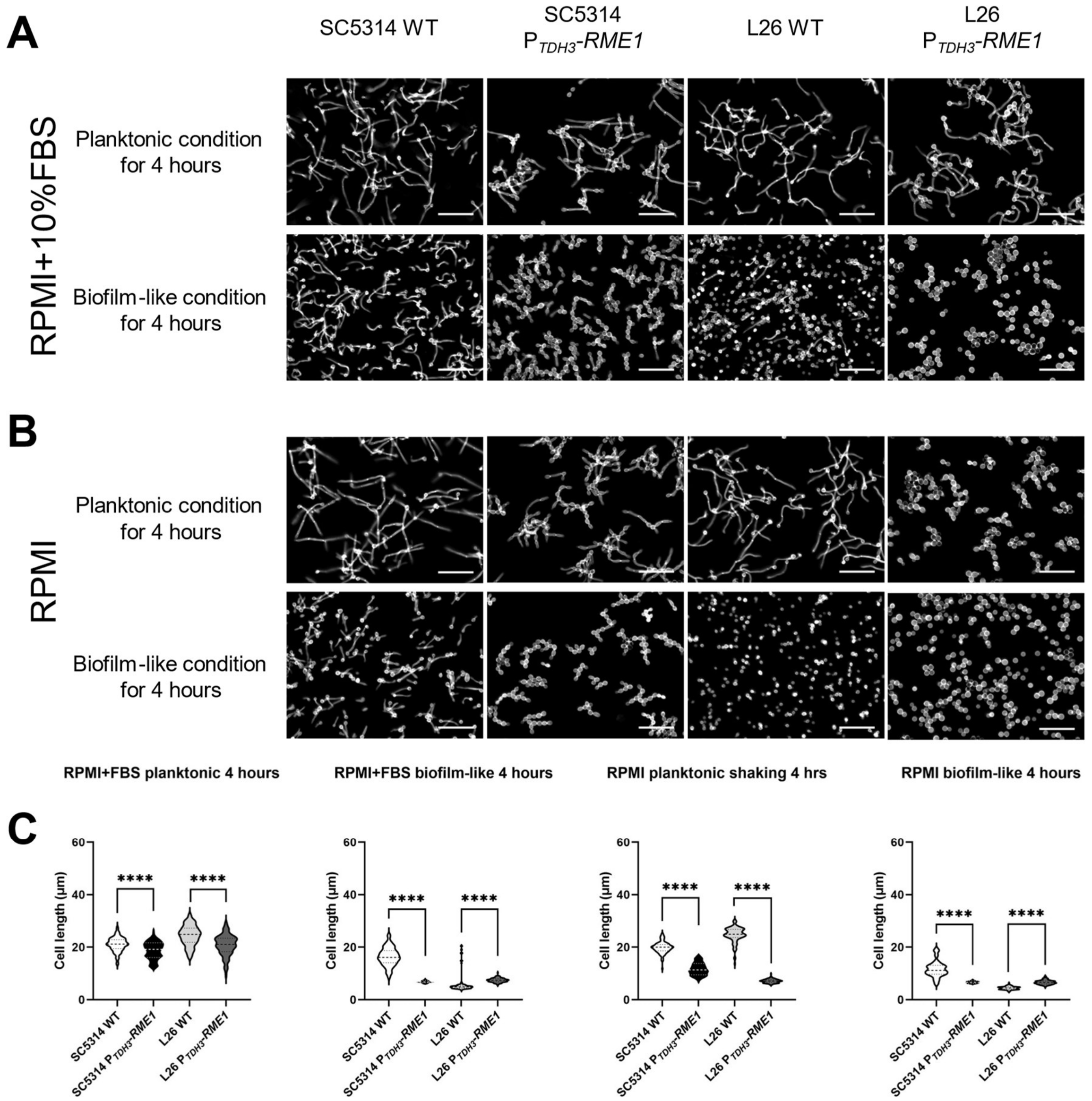

Figure S3. Biofilm and filamentation assays of *rme1*Δ/Δ mutants. A. Wild-type and *rme1*Δ/Δ strains in the backgrounds indicated were assayed for biofilm formation ability in RPMI +10% FBS at 37°C for 24 hrs in 96-well plates. Representative apical (left images) and side (right images) views are shown. The white scale bars indicate 100 µm. The graphs present biofilm volume measurement for biological triplicate samples. Statistical analysis was conducted using a one-way ANOVA, and asterisks denote statistically significant differences. ** p-value < 0.01, *** p-value < 0.001, **** p-value < 0.0001. **B.** Wild-type and *rme1*Δ/Δ strains in the SC5314 background were assayed for hypha formation ability in RPMI +10% FBS or RPMI (as indicated) at 37°C for 4 hrs. Representative images are shown. The white scale bars indicate 50 µm.

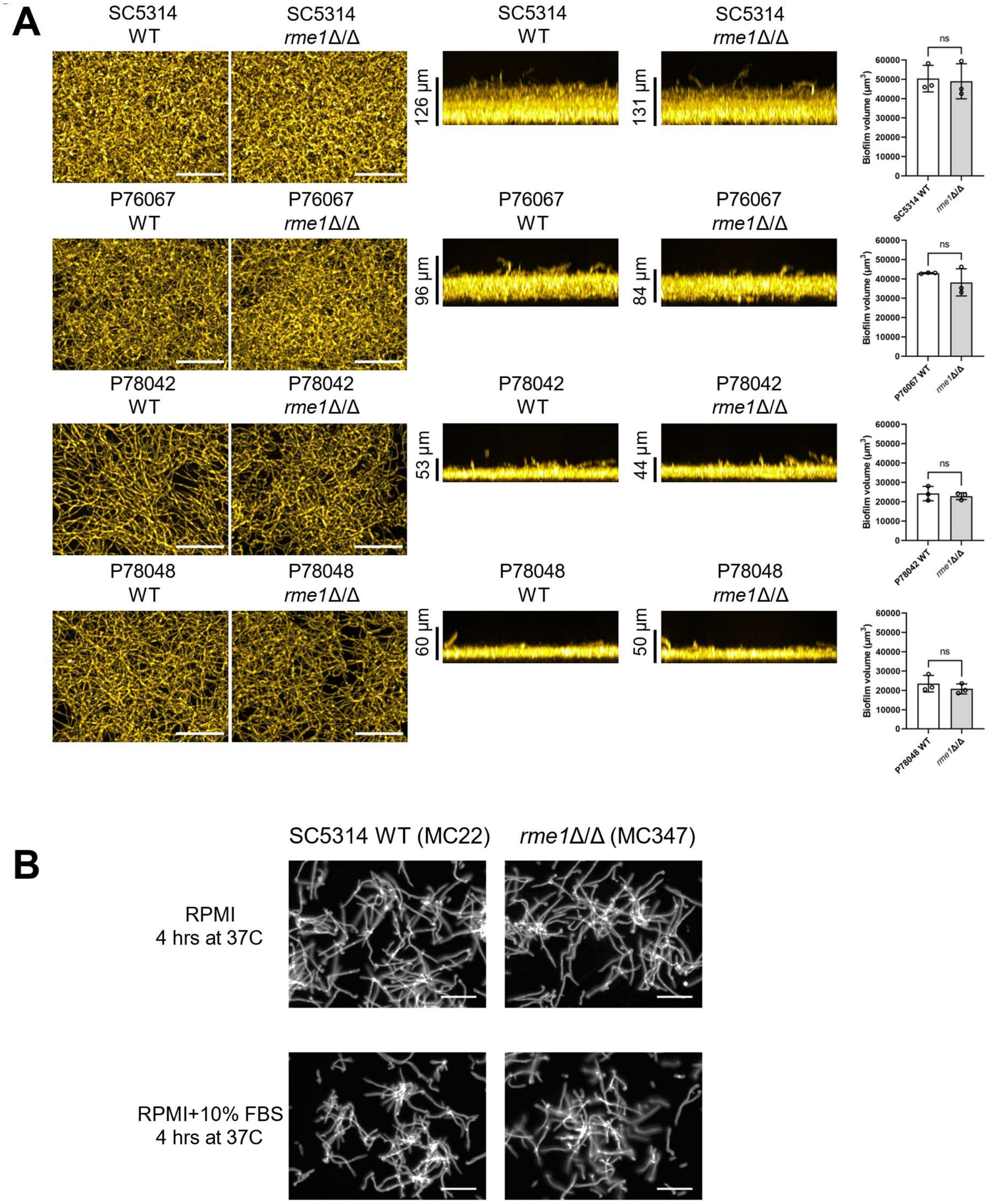

Figure S4. Impact of *rme1*Δ/Δ mutation on *efg1*Δ/Δ mutant biofilm formation. Biofilm assays were conducted on the wild type, *rme1*Δ/Δ and *efg1*Δ/Δ single mutants, and an *efg1*Δ/Δ *rme1*Δ/Δ double mutant in the SC5314 reference background. Biofilm formation was assayed in RPMI+10% FBS at 37°C for 24 hrs in 96-well plates. Representative side (above) and apical (below) views are shown. The white scale bars indicate 100 µm. The graphs show biofilm volume measurements for biological triplicates of each strain. Statistical analysis was conducted using a one-way ANOVA, and asterisks denote statistically significant differences. ** p-value < 0.01, *** p-value < 0.001, **** p-value < 0.0001.

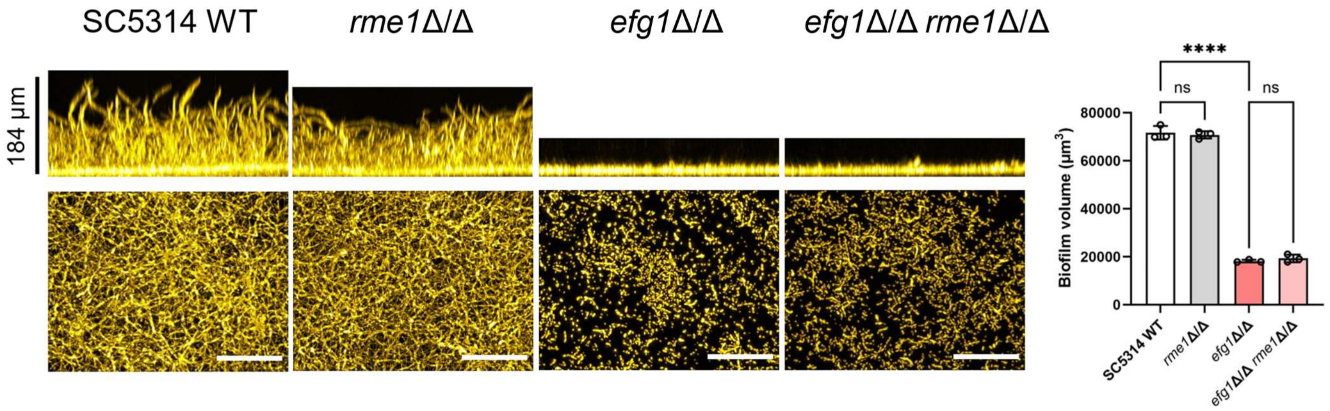

Figure S5. Impact of *rme1*Δ/Δ on *brg1*Δ/Δ mutant filamentation. Filamentation was assayed for the indicated SC5314-derived strains in planktonic conditions: RPMI or RPMI+10%FBS medium (as indicated), 4 hours, 37°C with vigorous shaking. Representative images are shown. The white scale bars indicate 50 μm in length. Graphs show cell length measurements from at least 8 fields of view and 100 cells. Statistical analysis was conducted using a one-way ANOVA, and asterisks denote statistically significant differences. ** p-value < 0.01, *** p-value < 0.001, **** p-value < 0.0001.

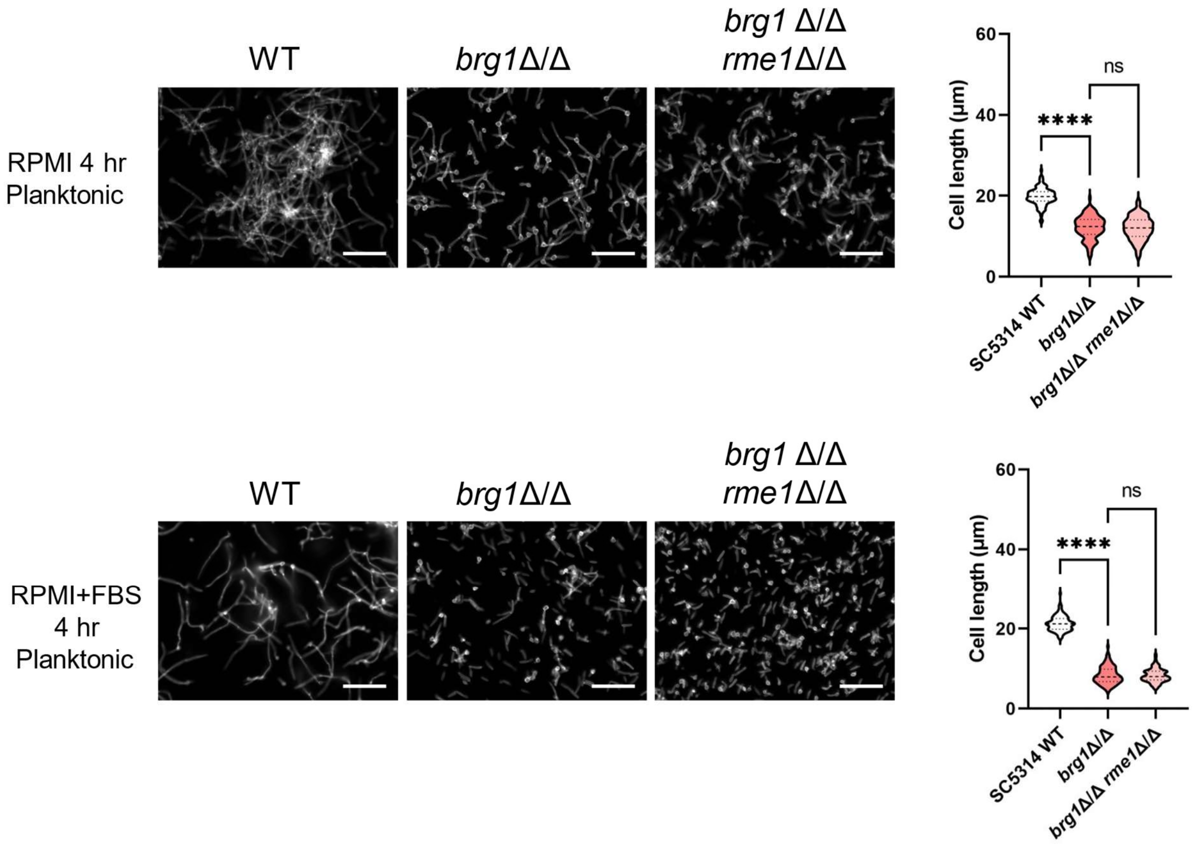

Figure S6. RME1 RNA levels under planktonic and biofilm-like conditions. Wild-type SC5314 and its *brg1*Δ/Δ mutant were grown in RPMI at 37 ̊C for 30 hours under planktonic or biofilm-like conditions. RNAs of three independent biological samples were extracted for qPCR determination. *RME1* mRNA levels were normalized to *SPA2* RNA levels. Relative gene expression was calculated using the threshold cycle ΔΔCT method. Statistical analysis was conducted using one-way ANOVA. *p-value < 0.05, ****p-value < 0.0001.

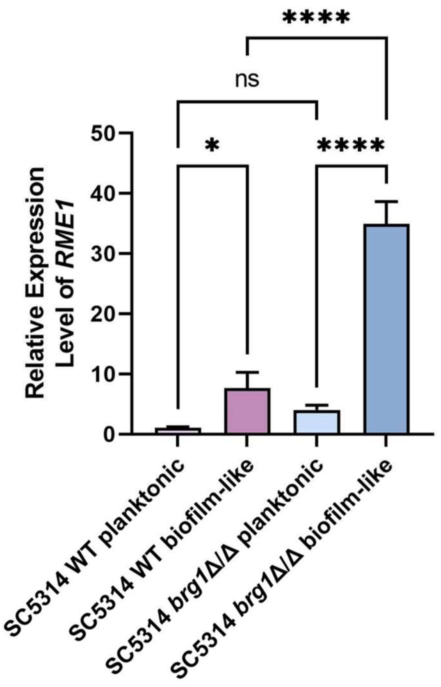

Supplement: Supplemental figures — Figures S1 to S6. [file mbio.01872-24-s0002.pdf]
